# Supplementary material for: Self-Protection versus Fear of Stricter Firearm Regulations: Examining the Drivers of Firearm Acquisitions in the Aftermath of a Mass Shooting
Source: Patterns (N Y). 2020 Aug 11;1(6):100082. doi: 10.1016/j.patter.2020.100082 (PMC7660383; doi:10.1016/j.patter.2020.100082)
Supplement: Document S1. Supplemental Experimental Procedures, Figures S1–S5, and Tables S1 and S2 [file mmc1.pdf]

PATTER, Volume *1*

Supplemental Information

Self-Protection versus Fear of Stricter Firearm Regulations: Examining the Drivers of Firearm Acquisitions in the Aftermath of a Mass Shooting

Maurizio Porfiri, Roni Barak-Ventura, and Manuel Ruiz Marín

Summary: Supplementary Information contains legal environment and population data, a year-by-year cluster analysis of States, a comparison between different definitions of firearm-related legal environments, statistical details about the time-series of media output, and the validation of the approach on a synthetic data set.

S1. Legal environment and population data

Figure S1 shows data on law restrictiveness and population in the form of two histograms. Note that the two histograms are independently ordered according to the value of the variable they represent.

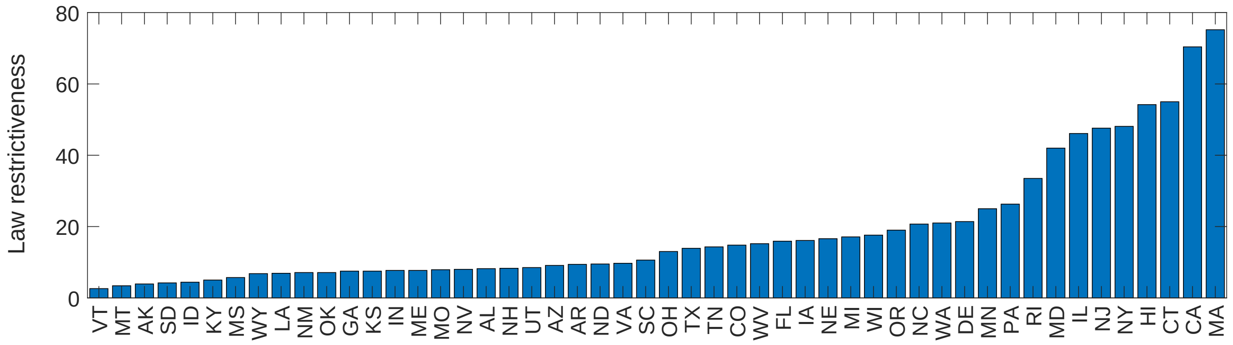

(a) Legal environment

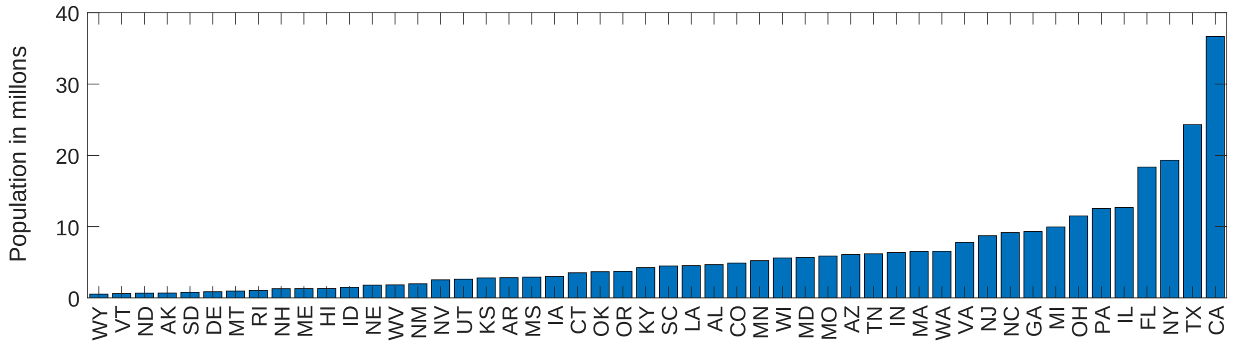

(b) Population

Figure S1: Law restrictiveness index, scored from 0% to 100%, and population in the US, averaged from 1999 to 2017.

S2. Year-by-year cluster analysis of States

Figure S2 shows the time-series of law restrictiveness for the 48 States included in our study, scored as the fraction of the 133 firearm safety laws that were in effect at any given year between 1999 and 2017. The time-series of each State is colored according to whether the State was scored as restrictive (red) or permissive (green) from the analysis in the main text, which was based on the average fraction of these laws that were in effect from 1999 to 2017.

As evident from the figure, changes in the legal environment across the Nation are secondary, whereby the time-series are always clustered in two groups. More precisely, the application of the k-means clustering algorithm year-by-year indicated that States group equivalently to the main text from 1999 to 2010. From 2011 to 2017, the algorithm would assign Rhode Island to the permissive group. Notably, the population of

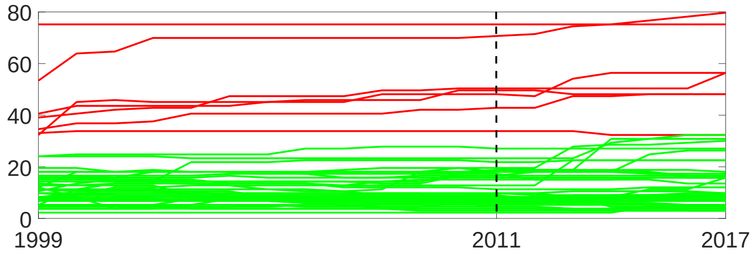

Figure S2: Time-series of law restrictiveness from 1999 to 2017, for the 48 States considered in the main text (all States, excluding Connecticut and Hawaii). Time-series are color coded according to their original assignment to the restrictive (red) or permissive (green) groups. The year 2011 is marked with a dashed line to indicate that a cluster analysis performed in any year in the range 2011 $-$ 2017 would lead to Rhode Island be assigned to the permissive group.

Rhode Island is much smaller than any the other State in the restrictive group, thereby causing a negligible change in the fraction of the population living in restrictive versus permissive States.

S3. Comparison between definitions of legal environments

We compared our definition of law restrictiveness with the one proposed by Reeping et al.,[1] which was based on the 1998-2015 edition of the Traveler's Guide to the Firearms Laws of the Fifty States. This report serves as a reference guide for firearm owners traveling between States: States with a rating of zero are completely restrictive and those with rating of one hundred are completely permissive. As detailed by Reeping et al.,[1] the conditions that the report takes into consideration to rate the States are more than 13, spanning from standard firearms ownership and permit requirements to whether it is possible to keep a firearm in one's vehicle at colleges and K-12 schools.

Figure S3 compares our metric of law restrictiveness of the firearm-related legal environment of a State (horizontal axis) with the score by Reeping et al.[1] (vertical axis), extracted from their supplementary figure by manually taking the medians of each of the boxplots. Excellent agreement between the metrics can be evidenced from the data, with an R^2 of 0.821.

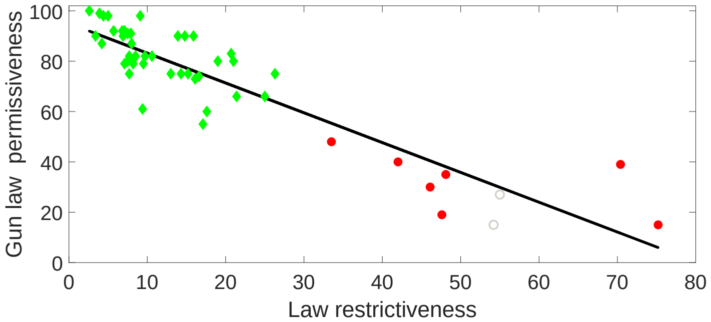

Figure S3: Comparison between the proposed measure of law restrictiveness (from 0% to 100%; 0% being completely permissive and 100% completely restrictive) and the law permissiveness score proposed by Reeping et al.[1] (from 0 to 100; 0 being completely restrictive and 100 completely permissive). Each marker corresponds to one of the 50 States and the color-coding is the same as in the main text (red circle: one of the seven States in the restrictive group; green diamond: one of the 41 States in the permissive group; and open grey circle: one of the two States that are excluded from the study due to lack of data). A regression line is also shown for clarity.

S4. Further statistical analysis of media output

Here, we provide further statistical details regarding the ten time-series of media coverage from the selected newspapers (Chicago Tribune, Los Angeles Times, New York Times, Times Picayune, and Wall Street Journal). First, we confirmed that the time-series could be treated as stationary by performing the augmented Dickey–Fuller test on each of them. The results of the analysis rejected the null hypothesis of unit root with $p < 0.001$.

Second, we examined the correlation between each pair of time-series pertaining to the same type of media coverage. Tables S1 and S2 report the values of the Pearson correlation coefficients for each of pair of media coverage of shootings and firearm control, respectively. Although all the coefficients are positive, they vary over a wide range from 0.007 to 0.899, suggesting that the selected newspapers contribute different levels of coverage of shootings and mass shootings. This is especially evident when examining media output on firearm control, in which both the Los Angeles Times and Times Picayune from California and Louisiana, respectively, neither correlate with each other nor with any other newspaper.

	Chicago Tribune	Los Angeles Times	New York Times	Times Picayune	Wall Street Journal
Chicago Tribune	1.000	0.644	0.873	0.106	0.899
Los Angeles Times	-	1.000	0.580	0.054	0.572
New York Times	-	-	1.000	0.134	0.876
Times Picayune	-	-	-	1.000	0.069
Wall Street Journal	-	-	-	-	1.000

Table S1: Pearson correlation coefficient between the time-series on media coverage of firearm control for the five newspapers.

	Chicago Tribune	Los Angeles Times	New York Times	Times Picayune	Wall Street Journal
Chicago Tribune	1.000	0.035	0.503	0.644	0.641
Los Angeles Times	-	1.000	0.007	0.030	0.031
New York Times	-	-	1.000	0.476	0.398
Times Picayune	-	-	-	1.000	0.624
Wall Street Journal	-	-	-	-	1.000

Table S2: Pearson correlation coefficient between the time-series on media coverage of shootings for the five newspapers.

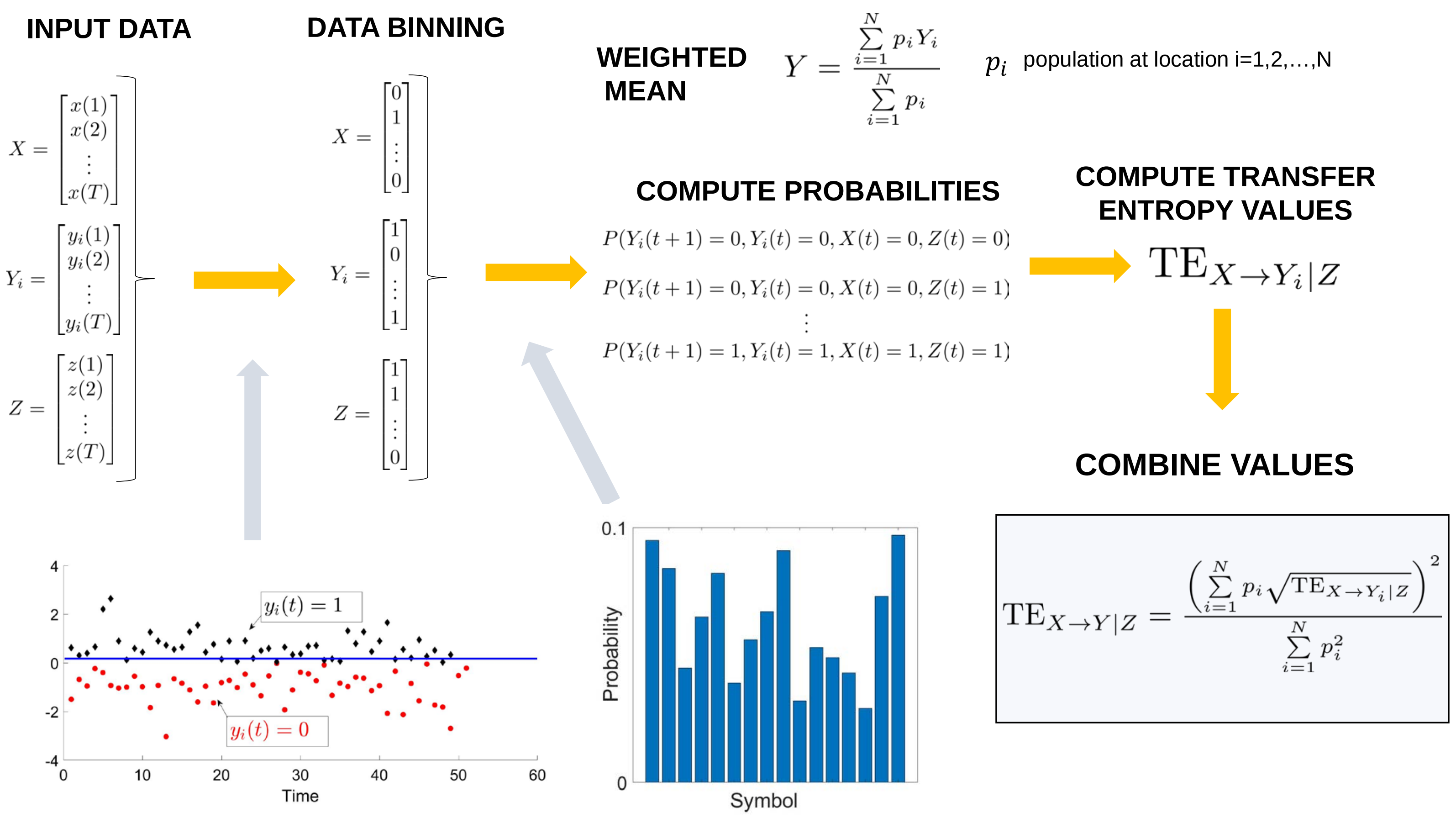

Figure S4: Sketch of the steps needed for transfer entropy computation. Starting from the time-series of all the processes of length $T = 228$, we compute binary versions by binning the data. Next, we estimate joint probability mass functions probabilities by simply counting the ones and the zeros, from which we calculate transfer entropy values for $i = 1, \dots, N$, with $N = 10$. Finally, the population values are utilized to calculate transfer entropy.

S5. Demonstration of the approach on synthetic data

To demonstrate the performance of the proposed causality analysis based on transfer entropy, we simulated a controlled experiment with synthetic data. Specifically, we considered the following linear autoregressive model, where X is the cause (similar to any of the media output or occurrence of mass shootings at the National level), Y is the effect, sampled at $N = 10$ different locations (similar to a pro capita background check at the State-level), and Z is a third independent process (acting as a potential confound):

$$X(t+1) = \alpha_X X(t) + \gamma_{XZ} Z(t) + \varepsilon_X \quad \text{(S1a)}$$

$$Y_i(t+1) = \alpha_Y Y_i(t) + \beta \gamma_{YXi} X(t) + \varepsilon_{Yi}, \quad i = 1, \dots N \quad \text{(S1b)}$$

where $\alpha_Y = 0.2$, $\alpha_X = 0.1$, $\gamma_{XZ} = 0.2$, $\gamma_{YXi} = 0.1$ for $i = 1, \dots, N/2$ and $\gamma_{YXi} = 0.3$ for $i = N/2+1, \dots, N$, and Z, ε_X, ε_{Yi}, with $i = 1, \dots, N$, are normal independent identically distributed with zero mean and unit variance, that is, $\mathcal{N}(0,1)$. The parameter β is used to tune the strength of the interaction.

S5.1. Inference of causal relationships

For each value of β from 0.1 to 2 with steps of 0.1, we simulated 100 processes through Eq. (S1) for 228 time-steps, equal to the length of the available time-series. For each simulation, we estimated the weighted sum of the square root of transfer entropy values $\mathrm{TE}_{X \to Y_i|Z}$ and $\mathrm{TE}_{Z \to Y_i|X}$, for $i = 1, \dots, N$, divided by the sum of the square of the populations. In the estimation, we assumed for each of the ten locations the following populations: $\{p_1 = 0.1, p_2 = 0.2, p_3 = 0.3, p_4 = 0.4, p_5 = 0.5, p_6 = 0.6, p_7 = 0.7, p_8 = 0.8, p_9 = 0.9, p_{10} = 1.0\}$. Fig. S4 illustrates the steps of the transfer entropy computation.

Figure S5 summarizes the accuracy of the inferences for 100 simulations. Therein, we specifically report the following quantities: true and false positive rates of rejections of the null hypothesis of no causality from X to Y, and true and false positive rates of failing to reject the null hypothesis of no causality from Z to Y. The permutation tests were carried out using 1,000 bootstrap realizations at a confidence level $\alpha = 0.050$. For example, a true positive rate of 90 on the causal link from X to Y indicates that in 90 out of 100 simulations, we rejected the hypothesis of no causality (that is, $p < 0.050$ in permutation tests). Similarly, a true positive rate of 90 on the causal link from Z to Y indicates that in 90 out of 100 simulations, we failed to reject the hypothesis of no causality (that is, $p \geq 0.050$ in permutation tests).

The results of the inference in Figure S5 offer compelling evidence in favor of the feasibility of the proposed approach to unfold causal relationships: not only is the method able to exactly discover the true causal link from X to Y for sufficiently large values of the coupling β (black markers), but also it is able to filter out the spurious interaction between X and Y for any choice of the parameter β (red markers).

S5.2. Verification of the weighting scheme for transfer entropy values

Next, we utilize the same example to illustrate the mathematical rationale for the chosen weighting scheme for transfer entropy values corresponding to the effect variables Y_i, for $i, \dots, N$. In the model in Eq. (S1), the effect variables represent pro capita background checks. Hence, computing the overall effect in the entire population requires multiplying each of them by the corresponding population. The resulting, normalized, variable is $Y = \left(\sum_{i=1}^{N} p_i Y_i\right) / \left(\sum_{i=1}^{N} p_i\right)$. Based on this aggregated variable, we calculate $\mathrm{TE}_{X \to Y|Z}$, which can be exactly computed from the classical expression for the entropy of a normal variable.[2] By recalling that the (differential) entropy of a normal variable G with variance σ_G^2 is $H(G) = \frac{1}{2}\log_2(2\pi e \sigma_G^2)$, where e is Nepero's number, we establish

$$\mathrm{TE}_{X \to Y|Z} = \frac{1}{2}\log_2\left(1 + \frac{\left(\sum_{i=1}^{N} p_i \gamma_{YXi}\right)^2 \sigma_X^2}{\sum_{i=1}^{N} p_i^2}\right) \quad \text{(S2a)}$$

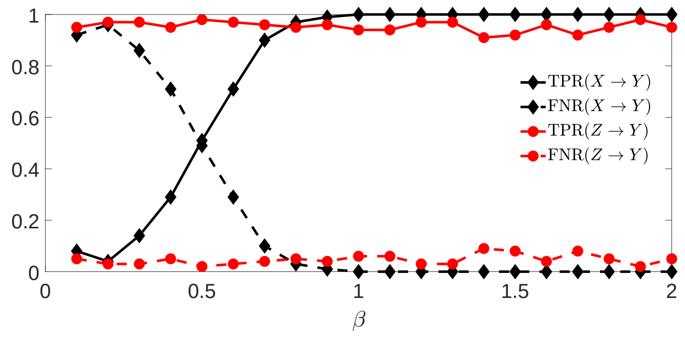

Figure S5: Results of the inference for a simulated data set, generated using Eq. (S1) and parameters reported in the text. Accuracy is assessed in terms of the rejection of the null hypotheses of no causality from X to Y and from Z to Y over 100 simulations. Specifically, we present the true positive rate of rejections of the null hypothesis of no causality from X to Y, $\mathrm{TPR}(X \to Y)$ (black solid line and diamond-shaped markers); false negative rate of rejections of the null hypothesis of no causality from X to Y, $\mathrm{FNR}(X \to Y) = 1 - \mathrm{TPR}(X \to Y)$ (black dashed line and diamond-shaped markers); true positive rate of failing to reject the null hypothesis of no causality from Z to Y, $\mathrm{TPR}(Z \to Y)$ (red solid line and circular markers); and false negative rate of failing to reject the null of hypothesis of no causality from Z to Y, $\mathrm{FNR}(Z \to Y) = 1 - \mathrm{TPR}(Z \to Y)$ (red dashed line and circular markers). The permutation tests were carried out using 1,000 bootstrap realizations at a confidence level $\alpha = 0.050$.

$$\mathrm{TE}_{X \to Y_i | Z} = \frac{1}{2} \log_2 \left(1 + \gamma_{YXi}^2 \sigma_X^2\right) \tag{S2b}$$

By multiplying both sides of the equations above by 2, taking the power of both sides, and carrying out a MacLaurin expansion for small values of transfer entropy, we obtain

$$\mathrm{TE}_{X \to Y | Z} \approx \frac{\left(\sum\limits_{i=1}^{N} p_i \sqrt{\mathrm{TE}_{X \to Y_i | Z}}\right)^2}{\sum\limits_{i=1}^{N} p_i^2} \tag{S3}$$

By applying the well-known Cauchy-Schwartz inequality, the numerator in Eq. (S3) can be bounded by $\left(\sum\limits_{i=1}^{N} p_i^2\right)\left(\sum\limits_{i=1}^{N} \mathrm{TE}_{X \to Y_i | Z}\right)$, such that

$$\mathrm{TE}_{X \to Y | Z} \lessapprox \sum_{i=1}^{N} \mathrm{TE}_{X \to Y_i | Z} \tag{S4}$$

This expression suggests that the value of transfer entropy in Eq. (S3) scales with N. Throughout the tables in the main text, we observe that transfer entropy values for restrictive States are, in fact, always a fraction of the corresponding values for permissive States.

REFERENCES

1. Reeping, P.M., Cerdá, M., Kalesan, B., Wiebe, D.J., Galea, S., Branas, C.C., (2019). State Gun Laws, Gun Ownership, and Mass Shootings in the US: Cross Sectional Time Series. BMJ 364, l542, `https://doi.org/10.1136/bmj.l542`.
2. Cover, T.M., and Thomas, J.A. (2006). Elements of Information Theory. (Wiley Series in Telecommunications and Signal Processing).
