# Supplementary material for: Self-Protection versus Fear of Stricter Firearm Regulations: Examining the Drivers of Firearm Acquisitions in the Aftermath of a Mass Shooting
Source: Patterns (N Y). 2020 Aug 11;1(6):100082. doi: 10.1016/j.patter.2020.100082 (PMC7660383; doi:10.1016/j.patter.2020.100082)
Supplement: Document S2. Article plus Supplemental Information [file mmc3.pdf]

Article

Patterns

Self-Protection versus Fear of Stricter Firearm Regulations: Examining the Drivers of Firearm Acquisitions in the Aftermath of a Mass Shooting

Graphical Abstract

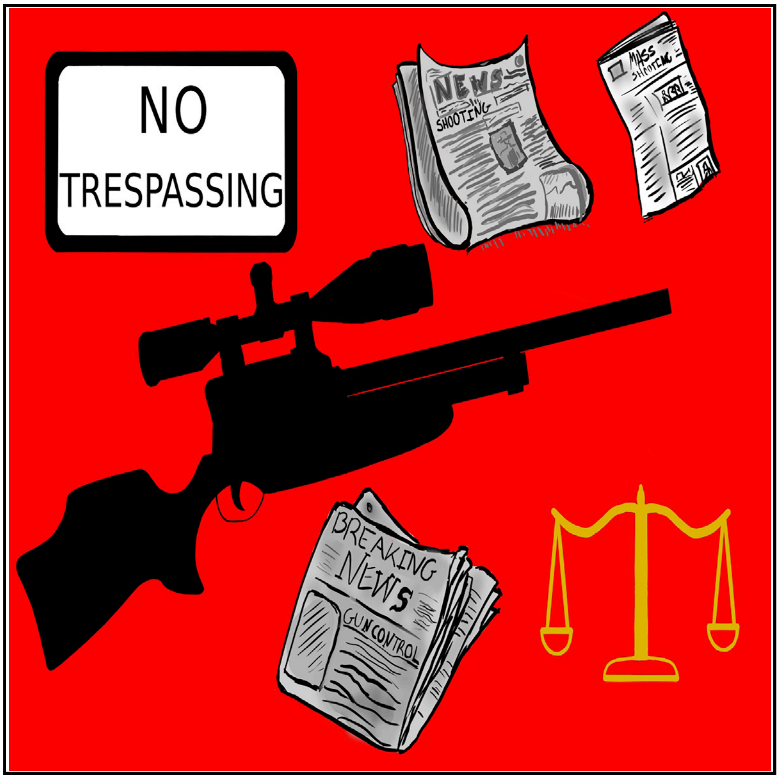

Highlights

- The drivers of firearm acquisition in America are studied in a State-level analysis
- An information-theoretic framework is developed to quantify causal relationships
- We detail the role of mass shootings, media output, and State-to-State interactions
- Fear of stricter firearm regulations can be a stronger driver than self-protection

Authors

Maurizio Porfiri, Roni Barak-Ventura, Manuel Ruiz Marín

Correspondence

mporfiri@nyu.edu

In Brief

Why are surges in firearm sales observed after a mass shooting? Are people concerned for their safety, as they worry that another mass shooting could take place? Or are they afraid that their access to firearms could be curtailed by stricter regulations? An information-theoretic framework is established to address these questions, utilizing State-level data on mass shootings and federal background checks, along with media coverage from different sources of shootings and firearm control.

Porfiri et al., 2020, Patterns *1*, 100082
September 11, 2020 © 2020 The Author(s).
https://doi.org/10.1016/j.patter.2020.100082

CellPress

Patterns

CellPress
OPEN ACCESS

Article

Self-Protection versus Fear of Stricter Firearm Regulations: Examining the Drivers of Firearm Acquisitions in the Aftermath of a Mass Shooting

Maurizio Porfiri,[1,2,3,4,*] Roni Barak-Ventura,[1] and Manuel Ruiz Marín[3]
[1]Department of Mechanical and Aerospace Engineering, New York University Tandon School of Engineering, Brooklyn, NY 11201, United States
[2]Department of Biomedical Engineering, New York University Tandon School of Engineering, Brooklyn, NY 11201, United States
[3]Department of Quantitative Methods, Law and Modern Languages, Technical University of Cartagena, Cartagena, Murcia 30201, Spain
[4]Lead Contact
*Correspondence: mporfiri@nyu.edu
https://doi.org/10.1016/j.patter.2020.100082

THE BIGGER PICTURE Surges in firearm acquisition after mass shootings have been widely documented in the United States for decades, but their underlying cause is yet to be fully elucidated. Do people purchase guns for self-protection, as they fear to be the next victims of a mass shooting? Or do they acquire guns because they fear firearms will be curtailed by upcoming policy actions? Or are they driven by both these fears? Answering these questions requires overcoming traditional correlation analysis through statistically principled approaches that can infer causal relationships from time-series. Here, we present a detailed information-theoretic analysis of State-level firearm acquisitions, which takes into consideration the location of mass shootings, State-to-State interactions, and firearm-related legal environment. Disentangling causation from correlation is critical in firearm research toward empowering policy makers with strong, objective support for effective policy solutions.

1 2 3 4 5 Mainstream: Data science output is well understood and (nearly) universally adopted

SUMMARY

Discovering causal mechanisms underlying firearm acquisition can provide critical insight into firearm-related violence in the United States. Here, we established an information-theoretic framework to address the long-disputed dichotomy between self-protection and fear of firearm regulations as potential drivers of firearm acquisition in the aftermath of a mass shooting. We collected data on mass shootings, federal background checks, media output on firearm control and shootings, and firearm safety laws from 1999 to 2017. First, we conducted a cluster analysis to partition States according to the restrictiveness of their firearm-related legal environment. Then, we performed a transfer entropy analysis to unveil causal relationships at the State-level in the Wiener-Granger sense. The analysis suggests that fear of stricter firearm regulations is a stronger driver than the desire of self-protection for firearm acquisitions. This fear is likely to cross State borders, thereby shaping a collective pattern of firearm acquisition throughout the Nation.

INTRODUCTION

Mass shootings are a critical public health issue in the United States (US), where more such events take place than anywhere else in the world.[1] Over the 15-year period from 1999 to 2013, the US has experienced more than 21 mass shootings per year, in which four or more people were killed in a single incident using exclusively firearms.[2] Even restricting the count of these incidents to only those in public spaces that are not associated with gang activities, we still mourn more than five mass shootings per year.[3] In the last 6 years alone, over 2,000 people have lost their lives in mass shootings.[4]

The ramifications of mass shootings extend beyond deaths, including long-term trauma and grief that touch not only those directly affected, but whole communities. Although mass shootings account for only a small percentage of firearm-related harms

This is an open access article under the CC BY license (http://creativecommons.org/licenses/by/4.0/).

in the US, which claimed almost 35,000 lives per year between 2009 and 2017,[5] they have a prominent role in shaping American public opinion about firearm regulation and, likely, increasing the Nation's appetite for firearms. Through the study of six mass shootings between 2000 and 2010, Wallace[6] determined an association between mass shootings and increased National firearm acquisition. Likewise, Studdert et al.[7] reported large increases in the number of handgun sales in California following the Sandy Hook Elementary School shooting in December 2012 and the San Bernardino shooting in December 2015. Similar evidence has been documented by Callcut et al.[8] examining firearm sales from 1996 to 2015 in California and by Liu and Wiebe[9] considering National firearm acquisitions from 1996 to 2016.

As proposed by Wallace[6] within the framework of appraisal theory, it is tenable that the reason for purchasing a firearm in the aftermath of a mass shooting is the desire for self-protection. In support of this proposition are data by Studdert et al.,[7] indicating that firearm acquisitions went up by 50% in the San Bernardino area after the Sandy Hook School shooting in Connecticut , while they increased by 85% after the local San Bernardino shooting. The fear of being a victim of a shooting has deep roots in Americans, as demonstrated by a number of surveys.[10,11] "Being the victim of a mass/random shooting" was ranked fourth among the fears of Americans in a 2014 survey by Chapman University (Orange, CA, US)[10] and almost a third of survey respondents reported to be afraid or very afraid of a "random mass shooting" in another study by Chapman University in 2017.[11]

Complementing and, sometimes, contradicting the explanation based on self-protection, some authors have proposed that increases in firearm acquisitions in the aftermath of mass shootings could be due to the fear of stricter regulations that may curtail access to firearms. For example, the 2016 analysis by the New York Times concluded that firearm sales systematically increased after each call for stricter gun controls,[12] and this very same proposition has been advocated by other authors.[13,14] The increase in firearm sales after the election of President Obama in 2008 and the subsequent drop after the election of President Trump in 2016 align with this proposition, as explained by Smith[15]: "President Barack Obama was the greatest gun salesman in America until Hillary Clinton ran to replace him. Sales soared to records because gun owners feared they would impose tougher gun restrictions. Now that a Republican endorsed by the National Rifle Association is in the White House, those supposed villains have disappeared. Sales of guns and ammo are falling, right along with the stocks of gun makers."

These two competing explanations, self-protection versus fear of stricter firearm regulations, were recently examined by Stroebe et al.[16] through a survey of gun owners and non-owners, conducted immediately before and after the Orlando shooting in June 2016. The authors expected to offer evidence in favor of any of the two explanations by contrasting responses of the two groups of survey respondents. Should self-protection be the driver of the increase in firearm sales, they would have expected to register purchases by non-owners; whereas purchases by owners would support the explanation of fear of stricter firearm regulations. Despite the merit of the study and the large pool of participants, findings from the authors were not conclusive. The explanation advocated by the authors is that the responses gathered by the study were those of "a vast majority of Americans to the Orlando mass shooting," while "the people responsible for the increase in background checks (a proxy of firearm acquisition) are of an atypical minority, too small to have a significant impact on our findings."

An alternative approach to address this dichotomy was established by Porfiri et al.[17] Therein, a multivariate, information-theoretic approach takes as input the National time-series of federal background checks (used as a proxy for firearm acquisition), incidence of mass shootings throughout the Nation, and media output on either shootings or firearm control (encapsulating the exposure of the population to fear-eliciting stimuli of either personal safety or stricter firearm regulations, respectively). Through the information-theoretic concept of transfer entropy,[18] the authors successfully unveiled cause-and-effect relationships among these variables. Causality is intended in the Wiener-Granger sense,[19,20] so that knowledge about the present value of a time-series (cause) improves the prediction of the future of another time-series from its present (effect).

The approach by Porfiri et al.[17] uncovered a causal relationship between media output on firearm control and background checks at the National level, thereby supporting the proposition that increases in firearm acquisitions are related to fear of stricter firearm regulations. In State-level analyses, the authors found that the restrictiveness of firearm control policies moderated the strength of this link: the less restrictive a State's legal environment is, the stronger the link will be. Possibly, this is because the fear of new regulations may be more justified in States where there has historically been less action on firearm regulations. At the same time, the study failed to identify a causal relationship between media output on shootings and National background checks, which would have offered evidence in favor of the explanation based on self-protection.

While offering a first step toward the study of Wiener-Granger causal relationships in firearm research, the effort by Porfiri et al.[17] did not fully clarify the specific role of self-protection and fear for stricter firearm regulations on firearm acquisition. Although the study indicated that the occurrence of mass shootings did not cause firearm acquisitions at the National level, it did not elucidate the existence of such a link at the State-level. Should the self-protection explanation hold true, it could be tenable to propose a differential response of each State, depending on their recent history of local mass shooting events. The State that suffered the most recent mass shooting may be more likely to experience an increase of firearm acquisitions, compared with the rest of the Nation.

Another factor that was not considered by Porfiri et al.[17] was State-to-State interactions, whereby the entire State-level analysis regarding the moderating role of the firearm-related legal environment was conducted under the premise that firearm acquisitions in each State are independent of those in other States. The accuracy of this hypothesis is yet to be tested, but empirical evidence from other fields of investigation in public health may suggest otherwise. Firearm acquisition within a State may be driven by firearm acquisition in bordering States, rather than being the result of isolated decision-making. For example, previous research by Abaid et al.[21] demonstrated a strong interaction in motor-vehicle deaths among neighboring States, which may be explained by the composition of the transportation infrastructure and the legal environment in the US. Likewise, Gallos et al.[22]

CellPress
OPEN ACCESS

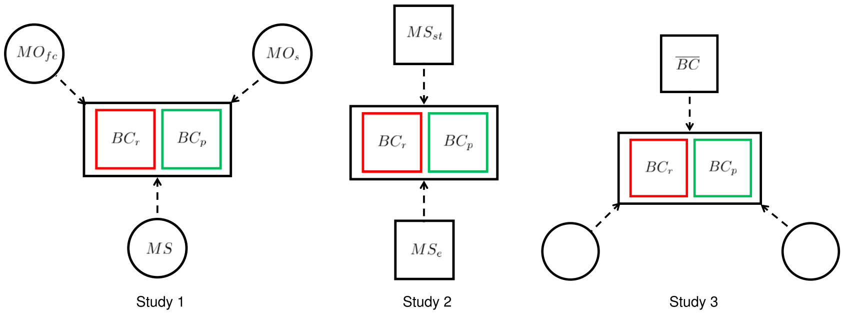

Figure 1. Graphical Representation of the Three Studies

For a Figure360 author presentation of this figure, see https://doi.org/10.1016/j.patter.2020.100082.

Figure360▷

Dashed arrows indicate potential cause-and-effect relationships. Circles are used to depict Nation-level, common variables and squares refer to variables that change as a function of the State under scrutiny, namely: number of background checks in restrictive (BC_r) or permissive (BC_p) States, occurrence of mass shootings at the Nation-level (MS), media output on firearm control (MO_{fc}), media output on shootings (MO_s), local mass shootings (MS_{st}), mass shootings that took place in any other State (MS_e), and number of background checks in neighboring States ($\overline{BC}$). Note that two variables in study 3 are deliberately left unnamed: study 1 will help identify the most influential variables for the analysis.

indicated the emergence of collective dynamics underlying obesity prevalence in the US, which may be related to similarities in the economic activity of supermarkets, food stores, and food services throughout the Nation.

Here, we apply a data science methodology to examine the explanations based on self-protection and fear of stricter firearm regulations, overcoming the limitations of the approach proposed by Porfiri et al.[17] Our approach is based on a granular, information-theoretic analysis of State-level firearm acquisitions, which takes into consideration the location of mass shootings, State-to-State interactions, and firearm-related legal environment.

First, we classify States on the basis of their firearm-related legal environment as "restrictive" or "permissive." Then, we perform three sequential studies (studies 1, 2, and 3; Figure 1) to explore the causal effects of the occurrence of mass shootings, media output on firearm control and shootings, and State-to-State interactions on State-level background checks. Across all these studies, we assess causality in a Wiener-Granger sense through a transfer entropy analysis that systematically controls for indirect coupling, which may lead to spurious conclusions. Study 1 seeks to pinpoint the main drivers of firearm acquisition by quantifying the effect of the occurrence of mass shootings at the National level, media output on firearm control, and media output on shootings in restrictive and permissive States. Study 2 delves into the effect of the location of a mass shooting, by tracking the State in which a mass shooting event has occurred within the transfer entropy analysis. Study 3 addresses the influence of geographically neighboring States, by controlling for the main drivers that have emerged from study 1.

RESULTS

Cluster Analysis: Partitioning States Based on the Restrictiveness of Their Firearm-Related Legal Environment

Using a k-means algorithm,[23] we partitioned the US States into two groups depending on the value of their law restrictiveness index (data in the Supplemental Information, Figure S1). Seven States were grouped in the restrictive cluster and 41 in the permissive cluster; two States were excluded from the analysis since their background checks' time-series were regarded to be lacking (Figure 2). Of the 1999–2017 average US population in the 48 considered States, 90,695,223 were living in restrictive States and 206,813,859 in permissive States. Using this partition, we conducted the three studies in Figure 1.

Study 1: Determining the Main Drivers of Firearm Acquisition

As a first step in the information-theoretic analysis, we examined the influence of the occurrence of mass shootings at the Nation-level (MS), media output on firearm control (MO_{fc}), and media output on shootings (MO_s) on either the number of background checks in restrictive States (BC_r) or in permissive States (BC_p) (study 1, Figure 1). When testing for the influence of any of the three potential causes on the number of background checks, we conditioned on the other two variables to control for their effect on the interaction.

The transfer entropy analysis identified a significant influence of media output on firearm control on background checks in permissive and restrictive States (p = 0.002 and p = 0.041 in permutation tests, respectively; Table 1). All the other potential influences are indistinguishable from chance ($p \geq 0.174$ in permutation tests; Table 1), although to a different extent, whereby the conditional transfer entropy value for the effect of media output on shootings in restrictive States is considerably closer to significance than others.

Figure 3 illustrates the values of some of the conditional probabilities that are used to compute conditional transfer entropy values in Table 1. These results provide evidence that media output on firearm control has a stronger effect on background checks throughout the Nation, than either mass shootings or media output on shootings. Specifically, the probability to register an increase in the number of detrended and seasonally adjusted background checks (from negative to positive values) in response to a surge in media coverage of firearm control can be as high as 0.600. Interestingly, some of the highly populated States in the permissive group are characterized by large probability values, which are not observed in any of the highly populated restrictive States. Both Michigan and Texas have a null probability of increasing their background checks, while they increase to 0.500 in response to a surge in media output on firearm control.

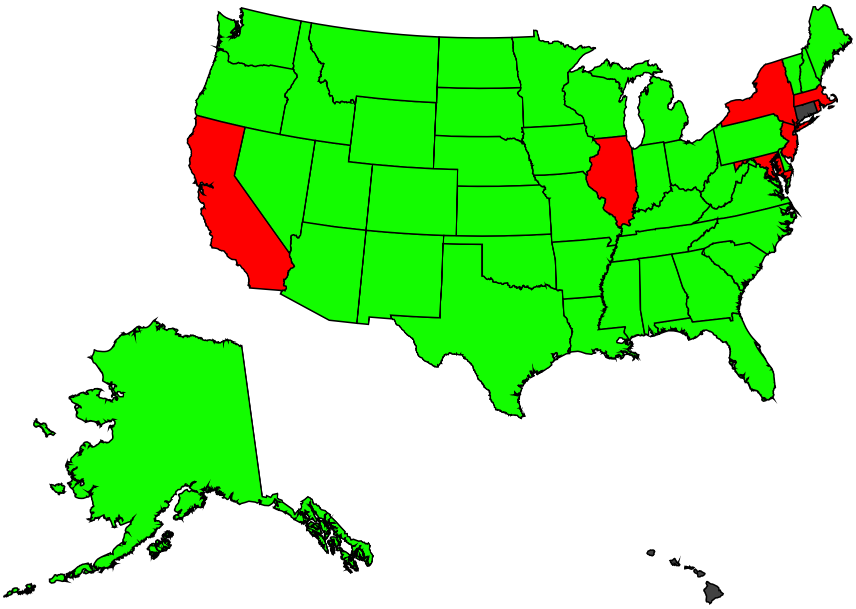

Figure 2. Cluster Analysis of US States according to their Firearm-related Legal Environment

The map identifies States with a restrictive (red: California, Illinois, Maryland, Massachusetts, New Jersey, New York, and Rhode Island) or permissive (green: Alabama, Alaska, Arizona, Arkansas, Colorado, Delaware, Florida, Georgia, Idaho, Indiana, Iowa, Kansas, Kentucky, Louisiana, Maine, Michigan, Minnesota, Mississippi, Missouri, Montana, Nebraska, Nevada, New Hampshire, New Mexico, North Carolina, North Dakota, Ohio, Oklahoma, Oregon, Pennsylvania, South Carolina, South Dakota, Tennessee, Texas, Utah, Vermont, Virginia, Washington, West Virginia, Wisconsin, and Wyoming) firearm-related legal environment. Gray color refers to the States that are excluded from the study due to lack of data (Connecticut and Hawaii).

Study 2: Delving into the Role of the Location of Mass Shootings

To delve into the specific role of mass shootings on the number of background checks, we performed an additional analysis in which we accounted for the location of the mass shootings. For each State, we separated the mass shootings time-series into two time-series: one time-series recording mass shootings that occurred in that particular State (MS_{st}), and the other pertaining to mass shootings that took place elsewhere in the Nation (MS_e). Through conditional transfer entropy, we studied the influence of each of these potential causes on the number of background checks in restrictive States or permissive States.

The analysis failed to identify a significant influence of mass shootings on the number of background checks, irrespective of the location of the event or the legal environment of the State ($p \geq 0.555$ in permutation tests; Table 2).

Study 3: Examining the Influence of Geographically Neighboring States

To account for State-to-State interactions, we examined the potential influence of geographically neighboring States on firearm acquisition in restrictive and permissive States. Specifically, we considered background checks in the n most proximal States of restrictive and permissive States ($\overline{BC}$), with n = 1, 3, 5, 7, and 9. In the analysis, we controlled for both media output on firearm control and media output on shootings, which were found to be the most critical drivers from Table 1.

For any choice of the number of neighbors, we determined a significant influence of background checks in neighboring States on the number of background checks in both permissive and restrictive States ($p \leq 0.046$ in permutation tests; Table 3). For any choice of the number of neighbors, we confirmed the influence of media output on firearm control on background checks in permissive States ($p \leq 0.006$ in permutation tests; Table 3). For restrictive States, a significant influence of media output on firearm control was registered in most of the cases (n = 1, 5, 9, $p \leq 0.040$ in permutation tests; Table 3); for other choices of the number of neighbors, influence did not reach statistical significance (n = 3, p = 0.077 and n = 7, p = 0.082 in permutation tests; Table 3). The influence of media output on shootings was not significant for any number of neighbors in permissive States ($p \geq 0.119$ in permutation tests; Table 3); in restrictive States, a significant influence was registered for the largest number of neighbors (n = 9, p = 0.049 and n = 1, 3, 5, 7, $0.071 \leq p \leq 0.241$ in permutation tests; Table 3).

DISCUSSION

Unraveling causal chains between firearm violence and availability is one of the most pressing methodological challenges to reduce the threat of firearm-related violence, as recognized by the National Research Council[24]: "Research on firearm violence that addresses the causal chain for tying a cause to an effect will provide important insights. This is especially true regarding research on gun availability and homicide. The widespread use of research study designs that have limited ability to study causality, like case-control and ecological studies, which aggregate data from sources and levels, poses challenges for interpretation among both researchers and policy makers."

This effort brings forward a granular, State-level analysis to clarify causal mechanisms underlying firearm acquisition in the US. Through a statistically principled approach grounded in information theory, we examined self-protection and fear of stricter firearm regulations as potential drivers of firearm acquisitions in the aftermath of mass shootings. The first step in the analysis entailed partitioning the States into two categories according to their firearm-related legal environment. Specifically, we utilized a k-means clustering algorithm using as input the fraction of firearm safety laws that were in effect in each State from 1999 to 2017. The partitioning resulted in two non-even groups, with restrictive States comprising about 15% of the Nation and 30% of its population. Such a partitioning was robust with respect to the selection of the time window and the definition of legal environments (further details in the Supplemental Information, Figures S2 and S3).

The analysis unfolded along three consecutive studies, designed to disentangle the effect of multiple factors that may

Table 1. Results from Study 1

Effect \ Cause	*MS*	MO_{fc}	MO_s
BC_r	0.0874	0.1450	0.1383
	(0.1502)	(0.1421)	(0.1568)
	p = 0.857	**p = 0.041**	p = 0.174
BC_p	0.4871	0.7166	0.5171
	(0.6527)	(0.6401)	(0.6565)
	p = 0.954	**p = 0.002**	p = 0.889

Influence of the occurrence of mass shootings at the Nation-level (*MS*), media output on firearm control (MO_{fc}), and media output on shootings (MO_s) on the number of background checks in States with restrictive (BC_r) or permissive (BC_p) firearm-related legal environment, according to the representation in Figure 1. Columns are potential causal variables and rows are effects. Influence is estimated through conditional transfer entropy, using Equation (S3) in the Supplemental Information. The numbers in parentheses denote the 95% quantile obtained from a permutation test with 20,000 surrogate time-series. A bold value indicates a significant positive conditional transfer entropy at $\alpha = 0.050$.

contribute to firearm acquisition in restrictive and permissive States through the application of transfer entropy on time-series. The first study examined the concurrent effect of the occurrence of mass shootings, media output on firearm control, and media output on shootings on the number of background checks in restrictive and permissive States. Despite methodological variations, the results of the study are in agreement with findings by Porfiri et al.[17] Different from the present effort, the analysis presented therein used mass shootings data from Mother Jones[3] and considered only two newspapers (the New York Times and Washington Post), without accounting for the simultaneous effect of two media outputs or partitioning States according to their legal environment.

From the first study, we conclude that media output on firearm control influenced the number of background checks in both permissive and restrictive States. Surges in media output on firearm control were associated with increases in background checks in highly populated permissive States, such as Michigan and Texas. Therein, we observed that an uptick in media coverage could reverberate in a dramatic growth in the probability that background checks could increase from 0% to 50%. Neither the occurrence of mass shootings nor media output on shootings were found to have an influence on background checks in permissive States, whereby an increase in either of these variable did not manifest into a robust increase in background checks.

This evidence seems to favor the proposition by Aisch and Keller,[12] Kegley,[13] and Naik[14] that firearm acquisitions are driven by fear of stricter firearm regulations more than self-protection against firearm violence. The significant influence of media output on firearm control on background checks suggests that people seek to purchase firearms when they fear that stricter regulations could soon be enforced in their State to curtail their access to firearms. Had the desire of self-protection been the main driver, we would have likely detected a significant influence on the number of background checks of either or both the occurrence of mass shootings and media output on shootings. Especially for permissive States, this was not the case, whereby transfer entropy values were far below the cutoff for statistical significance.

Obviously, failing to reject the null hypothesis of independence between two time-series does not allow to conclude that the two time-series are, in fact, independent. Hence, no conclusions should be drawn regarding the validity of the explanation based on self-protection, without incurring in the risk of a type I error, that is, of a false negative.[25] In particular, we should not exclude the possibility that media output on shootings plays some role on background checks in restrictive States. As a result, through the present analysis, it is difficult to eliminate the prospect of a synergistic effect of self-protection theory and fear of stricter regulations underlying firearm prevalence in restrictive States.

The specific role of the occurrence of mass shootings on background checks, however, seems to be secondary. In fact, the second study failed to offer statistical evidence in favor of an effect of mass shootings on background checks in both permissive and restrictive States. Even when focusing on the specific mass shootings that occurred in a State, we did not identify a causal mechanism in favor of the self-protection theory. We cannot exclude that the limited length of the time-series could have masked hidden causal mechanisms, but the modest transfer entropy values suggest that the occurrence of mass shootings was not a salient driver of firearm acquisition. These claims are in partial disagreement with observations by Wallace,[6] but there are several methodological differences between the two approaches that challenge the comparison of the predictions. There are differences in the datasets and statistical analysis: six mass shootings were studied by Wallace,[6] while 87 events were considered herein; Google Searches were utilized in Wallace,[6] while newspaper articles were used herein; and the analysis by Wallace[6] assumes linearity among the variables, which is partially obviated by the use of an information-theoretic approach.

The third study addressed an untapped area of research in the context of firearm acquisition, which is the quantification of State-to-State interactions. We demonstrated a significant influence of the number of background checks in neighboring States on background checks in both permissive and restrictive States. Such a prediction is based on aggregating the overall effect of neighboring States into a single, State-specific time-series that encapsulates the overall tendency to acquire firearms in geographically close regions. The classical theory of policy diffusion[26] could help frame this finding, whereby interactions between States are likely to affect firearm-related policy making.[27–29] Particularly relevant is the common belief in policy diffusion that some States consistently act as innovators of new policies,[26] while other States follow their footsteps and emulate successful policies. In this sense, acquisitions in neighboring States could signal probable changes in local firearm regulation that might diffuse across State borders. Whether or not such a perception translates into the spill-over of legislation across States is yet to be documented.[30]

An alternative explanation for the observed link between the number of background checks in neighboring States could be sought in the theory of contagion of mass shootings, which posits that the occurrence of a mass shooting in a particular State might quickly trigger other mass shootings in the same State or in other States.[31] Based again on self-protection, one may propose that people would seek to acquire firearms as they anticipate mass shootings to occur in their State. However,

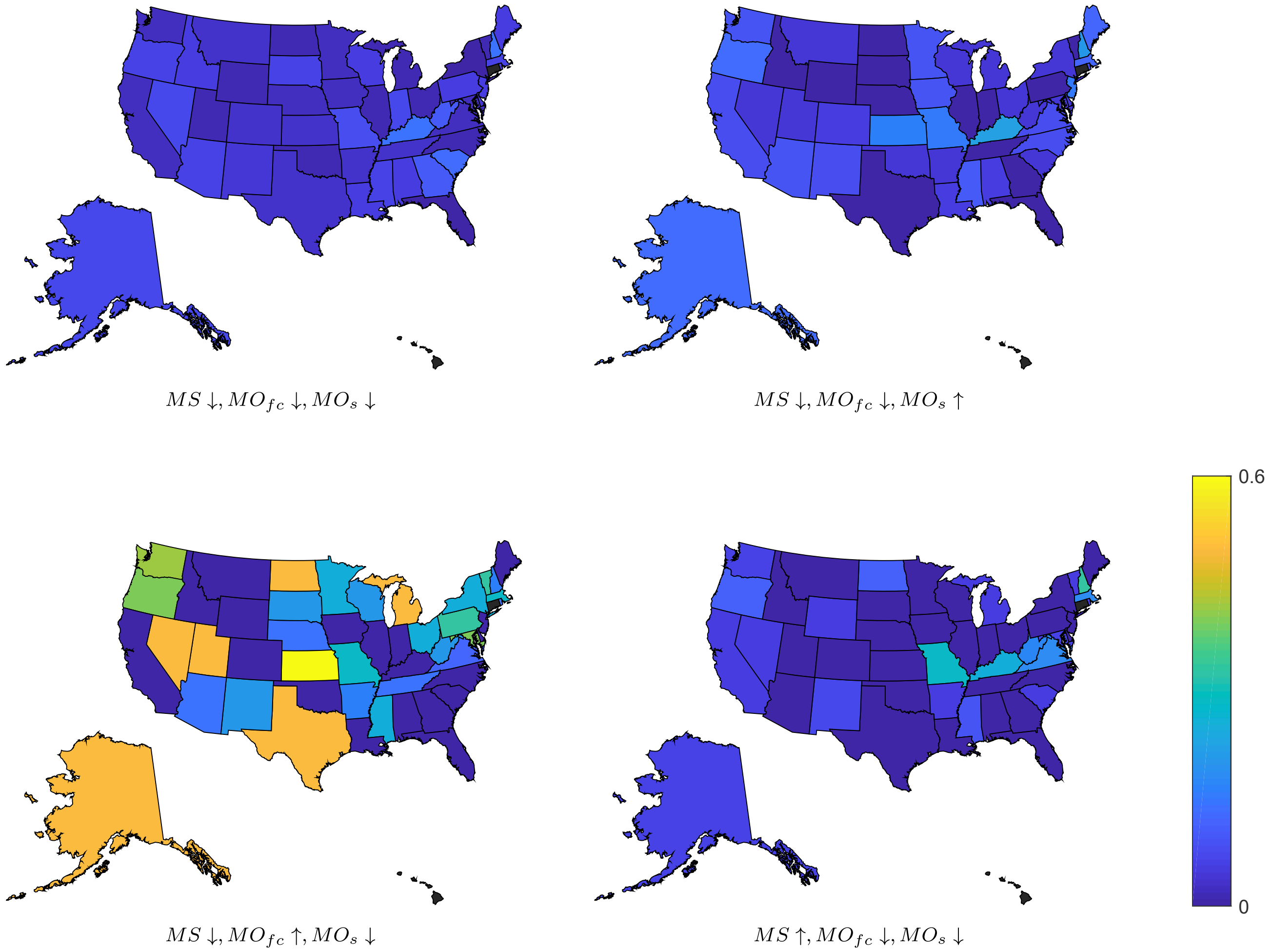

Figure 3. Visualization of the Potential Causes of Firearm Acquisitions from Study 1

The maps display the conditional probability that the binary time-series of background checks (*BC*) at time $t+1$ is ↑, conditioned to the event that is equal to ↓ at time t and that the binary time-series of mass shootings at the Nation-level (*MS*), media output on firearm control (MO_{fc}), and media output on shootings (MO_s) are equal to the indicated value at time t. Gray color refers to the States that are excluded from the study.

the lack of an influence of the occurrence of mass shootings on the number of background checks in permissive and restrictive States does not support this possibility.

Interestingly, for any selection of the number of neighbors, we confirm evidence from the first study that background checks in permissive States are largely driven by media output on firearm control. For restrictive States, accounting for interactions between States brings better to light the potential synergistic effect of the two theories. Specifically, we register comparable values of conditional transfer entropy for media output on firearm control and media output on shootings, wavering at the threshold of statistical significance. On the one hand, this evidence further supports the proposition that firearm prevalence in restrictive States is driven by the interaction between multiple mechanisms. On the other hand, it suggests that fear of stricter firearm regulations could be stronger in permissive States, whereby controlling for State-to-State interactions does not weaken the influence of media output on firearm control on background checks. People living in permissive States may be more likely to be driven by fear of firearm regulations in their decision to purchase a firearm, expecting that the legal environment in their State may soon become stricter.

The present data science methodology is not free of limitations. The use of binary representations for all the time-series cannot resolve fine details about the dynamics of the processes. However, increasing the complexity of the representation may reduce statistical power due to the relatively small size of the time-series of about 200 observations. The same limitation in the length of the time-series challenges the extension of the approach for the detection of causal links beyond those underlying the evolution of four processes. As a result, the present methodology cannot be used to tease out the most influential media sources or identify the most influential States in the Nation. Not only are the time-series short, but also their time-resolution is only at a monthly rate, which is not sufficient to avoid contemporaneous effects. We cannot exclude that two processes influence each other within the same month, thereby challenging the detection of a causal link through transfer entropy. Finally, the proposed strategy to weigh transfer entropy values of different States to obtain a single measure has some degree of arbitrariness. Although this weighting

CellPress
OPEN ACCESS

Table 2. Results from Study 2

Effect \ Cause	MS_{st}	MS_e
BC_r	0.0114	0.0158
	(0.0303)	(0.0487)
	$p = 0.589$	$p = 0.711$
BC_p	0.0473	0.1274
	(0.0794)	(0.1802)
	$p = 0.627$	$p = 0.555$

Influence of local mass shootings (MS_{st}) and mass shootings that took place elsewhere in the Nation (MS_e) on the number of background checks in States with restrictive (BC_r) or permissive (BC_p) firearm-related legal environment (BC_p), according to the representation in Figure 1. Columns are potential causal variables and rows are effects. Influence is estimated through conditional transfer entropy using Equation (S3) in the Supplemental Information. The numbers in parentheses denote the 95% quantile obtained from a permutation test with 20,000 surrogate time-series.

scheme is exact for a class of processes, its assumptions might be strained in the presence of strong and nonlinear interactions.

Beyond limitations associated with the data science methodology, the framing of the research and the data collection could also benefit from further research. First, the present approach offers a first categorization of the firearm-related legal environment through a single metric, but cannot assist in isolating the effectiveness of any particular law. Addressing the latter issue requires a different approach to causal analysis that is tailored to time-varying phenomena, which are instead filtered out in the present transfer entropy analysis. Second, mass shootings are only considered as binary events, without tracking their severity or any of the metrics that should be used to elucidate their etiology.[32] We should also mention that a universal definition of mass shootings has yet to be accepted by the community; even the number of victims used in defining the event may vary across databases.[33] Third, firearm acquisition is inferred through the number of background checks, which does not reflect all purchases (illegal and legal).[6] Finally, the assessment of media output is limited to articles appearing in newspapers, without accounting for the process of active information seeking by the public that could offer insight into the potential influence of the event.[34]

Data science methodologies to time-series analysis could beget new insight into firearm-related violence, which has been largely investigated through correlation analyses, linear regression techniques, and evidence-based inferences. Through the application of information-theoretic tools, we offered compelling evidence in favor of the theory that fear of stricter firearm regulations is a driver of firearm acquisitions and showed an interaction between States with respect to firearm acquisition. Our data science methodology is based on a particular notion of causality, grounded in the seminal work of Wiener and Granger.[19,20] In the Wiener-Granger sense, causality is measured from the improved statistical predictability of a process due to knowledge about other processes. While this notion of causality can be quantitatively examined from available observations, it is neither based on experimental manipulations nor does it beget a mathematical model to carry out what-if analyses. Designing experimental studies to validate our claims, while formulating mathematical models for the discovered relationships, should be the objective of future research.

EXPERIMENTAL PROCEDURES

Resource Availability

Lead Contact

Maurizio Porfiri, PhD, mporfiri@nyu.edu.

Materials Availability

This study did not generate any materials.

Data and Code Availability

Datasets and MATLAB scripts and codes can be downloaded from the GithHub repository of the Dynamical Systems Laboratory at New York University: https://github.com/dynamicalsystemslaboratory/Causes-of-firearm-acquisition. Original data have also been deposited to Mendeley Data (https://doi.org/10.17632/pn7scdrzx2.1).

Information-Theoretic Framework

Causal influence between variables was studied within an information-theoretic framework, where causality should be intended in a Wiener-Granger sense. As explained by Bressler and Seth:[35] "Causality in the Wiener-Granger sense is based on the statistical predictability of one time-series that derives from knowledge of one or more others." From raw temporal observations of a set of processes, information theory enables the inference of causality in the Wiener-Granger sense between any of the processes, without the need of an underlying mathematical model. In principle, the approach is applicable to linear and nonlinear interactions, and statistical tests could be carried out in a completely non-parametric way.[18] Across a range of applications where mathematical models are neither available or difficult to develop, researchers have clarified causal links in complex systems. For example, information theory is routinely applied to study the brain,[36] climate networks,[37] and animal groups.[38]

The premise of an information-theoretic approach is the notion of "entropy," as a fundamental measure of the uncertainty encoded in a random variable. Given a discrete random variable X, its entropy $H(X)$ is equal to

$$H(X) = -\sum_{x\in\mathcal{X}} P(X=x)\log_2 P(X=x), \qquad \text{(Equation 1)}$$

where $\mathcal{X}$ is the sample space of the variable, $P(\cdot)$ indicates probability, and the logarithm is taken in base 2 to measure entropy in bits. For example, given a Bernoulli random variable with probability p, the entropy is equal to $-p\log_2 p-(1-p)\log_2(1-p)$; entropy approaches 0 when the variable becomes deterministic ($p\rightarrow 0$ or 1) and is maximized when it is the most difficult to predict the outcome of the random variable ($p = 1/2$).

Working with two processes, we can use the notion of entropy to investigate Wiener-Granger causal influence of one process on the other. In particular, a cause-and-effect relationship between two processes, in the Wiener-Granger sense, implies that it is possible to improve the extent to which we can predict the future of one of the processes (effect) from its present due to additional knowledge about the present of the other process (cause). More specifically, given two discrete-time stationary processes X and Y, transfer entropy from X to Y, $\mathrm{TE}_{X\rightarrow Y}$, is equal to[18]

$$\mathrm{TE}_{X\rightarrow Y} = H(Y(t+1)|Y(t)) - H(Y(t+1)|Y(t),X(t)). \qquad \text{(Equation 2)}$$

If X does not encode useful information to predict Y, conditioning on $X(t)$ does not reduce the uncertainty of $Y(t+1)$, thereby leading to zero transfer entropy. The computation of transfer entropy in Equation (2) does not require the specification of any mathematical model; however, for Gaussian processes, transfer entropy becomes equivalent to the log likelihood statistic in Granger causality.[39] Specifically, transfer entropy corresponds to the logarithm of the ratio between the variance in the null regression model (Y independent of X) and the variance in the causal regression model.

In its basic incarnation, transfer entropy is designed to unveil Wiener-Granger causality between two processes. Dealing with multiple processes requires controlling for indirect coupling that might lead to spurious results. For example, given three processes X, Y, and Z in which X influences Z and Z influences Y, one might discover non-zero transfer entropy from X to Y, which, in turn, would prompt the inference of an erroneous cause-and-effect relationship. To mitigate these potential confounds in the discovery of a causal interaction between two processes, one should condition on all

Table 3. Results from Study 3

Effect \ Cause	$\overline{BC}$	MO_s	MO_{fc}	No. of Neighbors
BC_r	0.1416	0.1323	0.1383	$n = 1$
	(0.1404)	(0.1567)	(0.1330)	
	**p = 0.046**	p = 0.241	**p = 0.035**	
BC_p	0.9974	0.6270	0.7173	$n = 1$
	(0.5091)	(0.6492)	(0.6204)	
	**p < 0.001**	p = 0.119	**p < 0.001**	
BC_r	0.1784	0.1424	0.1374	$n = 3$
	(0.1243)	(0.1541)	(0.1447)	
	**p = 0.001**	p = 0.120	p = 0.077	
BC_p	0.8608	0.5172	0.6510	$n = 3$
	(0.4499)	(0.6033)	(0.5963)	
	**p < 0.001**	p = 0.420	**p = 0.004**	
BC_r	0.2188	0.1598	0.1543	$n = 5$
	(0.1221)	(0.1654)	(0.1377)	
	**p < 0.001**	p = 0.071	**p = 0.018**	
BC_p	0.7862	0.5118	0.6376	$n = 5$
	(0.4448)	(0.5811)	(0.5822)	
	**p < 0.001**	p = 0.356	**p = 0.002**	
BC_r	0.2167	0.1427	0.1386	$n = 7$
	(0.1281)	(0.1654)	(0.1446)	
	**p < 0.001**	p = 0.210	p = 0.082	
BC_p	0.8178	0.5489	0.6477	$n = 7$
	(0.4614)	(0.6098)	(0.5990)	
	**p < 0.001**	p = 0.327	**p = 0.001**	
BC_r	0.2214	0.1563	0.1444	$n = 9$
	(0.1170)	(0.1556)	(0.1416)	
	**p < 0.001**	**p = 0.049**	**p = 0.040**	
BC_p	0.8494	0.5731	0.6482	$n = 9$
	(0.4533)	(0.6155)	(0.6056)	
	**p < 0.001**	p = 0.186	**p = 0.006**	

Influence of the number of background checks in neighboring States ($\overline{BC}$), media output on firearm control (MO_{fc}), and media output on shootings (MO_s) on the number of background checks in States with restrictive (BC_r) or permissive (BC_p) firearm-related legal environment, according to the representation in Figure 1. The analysis is performed by varying the number of neighbors (n = 1, 3, 5, 7, and 9), whose background checks are aggregated in a single time-series encapsulating the effect of geographic neighborhood. When n = 1, only the closest State is included in the analysis, when n = 3 the three closest States are retained, and so on. Note that neighboring States are not classified according to their firearm-related legal environment, such that a permissive State may be interacting with restrictive States, and, likewise, a restrictive State may be interacting with permissive States. Columns are potential causal variables and rows are effects. Influence is estimated through conditional transfer entropy, using Equation (S3) in the Supplemental Information. The numbers in parentheses denote the 95% quantile obtained from a permutation test with 20,000 surrogate time-series.A bold value indicates a significant positive conditional transfer entropy at $\alpha = 0.050$.

the other processes in the computation of transfer entropy. The resulting version of transfer entropy is called conditional or partial transfer entropy.[18] In general, given a set of q potentially confounding processes $Z_1(t), \ldots, Z_q(t)$, we compute

$$\mathrm{TE}_{X\to Y|(Z_1,\ldots Z_q)} = H(Y(t+1)|Y(t), Z_1(t), \ldots, Z_q(t)) - H(Y(t+1)|Y(t), X(t), Z_1(t), \ldots, Z_q(t)). \quad \text{(Equation 3)}$$

In practical terms, seldom do we have access to exact probability distributions and we must rely on estimations that are based on time-series of finite length. The fundamental quantity that is needed for computing conditional transfer entropy is the joint probability distribution $P(Y(t+1), Y(t), X(t), Z_1(t), \ldots, Z_q(t))$, which can be estimated using simple plug-in frequency estimators upon binning the time-series. Given the length of the time-series, the need to accurately estimate this distribution limits the maximum number of confounding processes that can be examined at once.[40] Considering b bins for each of the time-series and q confounding variables, we are tasked with estimating $b^{(q+3)}$ probability values. Two hundred observations could be sufficient to perform the analysis for binary variables ($b = 2$) and two or less confounding variables ($q \leq 2$), but increasing the number of bins or the number of confounding variables would swiftly lead to having more probability values to estimate than available observations.

Data Collection

The data utilized herein regarding firearm legal environment, background checks, and population are in the Supplemental Information of Porfiri et al.[17] The geographic distance between the States was taken from Abaid et al.[21] Data on mass shootings and media output were collected as part of this effort to complement the database of Porfiri et al.[17]; all computer codes and datasets are available at https://github.com/dynamicalsystemslaboratory/Causes-of-firearm-acquisition. Below, we succinctly describe the dataset and the criteria adopted to originally compile it.

The restrictiveness of the legal environment of each State with respect to firearm regulation was evaluated by using data from the Firearm Laws Project website,[41] which contains an exhaustive database of 133 firearm safety laws in each of the 50 States from 1991 until 2017. Laws in the database pertain to different aspects of firearm safety distributed across 14 categories, such as prohibitions for people with high risks and domestic violence records, regulations on assault weapons and large-capacity magazines, and regulations limiting some types of ammunition. Law restrictiveness of each State was quantified as the fraction of these 133 laws that were in effect between 1999 and 2017. For example, Massachusetts scored the largest value of law restrictiveness of 75.2%, indicating that there were on average 100 laws between 1999 and 2017 out of the 133 included in the database. Likewise, Vermont had the lowest value of law restrictiveness of only 2.6%, meaning that it had on average only 3.5 laws in effect out of 133 in the database within the same time window. State-by-State law restrictiveness data are presented in the Supplemental Information (Figures S1 and S2). An equivalent assessment of law restrictiveness in the Nation would emerge from using the criteria recently proposed by Reeping et al.,[42] based on the 1998–2015 edition of the Travelers Guide to the Firearms Laws of the Fifty States, or scoring law restrictiveness on a yearly basis (further details in the Supplemental Information, Figure S3).

Population data were obtained from the website of the US Census Bureau and averaged from 1999 to 2017 (data in the Supplemental Information, Figure S1). Distance between States was measured from the geodesics between their centroids, as reported in Abaid et al.[21]

Data on the incidence of mass shootings were obtained from the Washington Post database,[43] which contains a list of mass shootings from August 1, 1966 to the present (June 8, 2020). The database was compiled by a group of the journal's researchers from data provided by criminologist Grant Duwe, Mother Jones,[3] and FBI homicide reports.[44] It consists of 176 shootings in which four or more people were killed, excluding shootings linked to robberies, drug-related crimes, and domestic events in private homes. For the purpose of this study, we considered the 87 events between 1999 and 2017. Inconsistencies in the reported dates of two mass shootings were found (Burns International Security shooting on September 8, 2001, and Su Jung Health Sauna shooting on February 21, 2012) and corrected when we compiled mass shooting occurrence at a monthly resolution. Table 4 shows all the 87 mass shootings, from 1999 to 2017 and Figure 4 reports them in the form of a time-series at the Nation level.

As a proxy of firearm acquisition in each of the 50 US States, we utilized the monthly federal weapons background check numbers.[45] The National Instant

CellPress
OPEN ACCESS

Table 4. Mass Shootings in the US from 1999 to 2017

Mass Shootings from 1999 to 2017		
New St. John Fellowship Baptist Church shooting, LA, 3/10/99	Columbine High School massacre, CO, 4/20/99	Albertson's supermarket shooting, NV, 6/3/99
Day-trading firms shooting, GA, 7/29/99	Wedgwood Baptist Church shooting, TX, 9/15/99	Xerox Engineering Systems shootings, HI, 11/2/99
Raddison Bay Harbor shooting, FL, 12/30/99	Mi-T-Fine Car Wash shooting, TX, 3/20/00	Mount Lebanon shooting, PA, 4/28/00
Edgewater Technology shooting, MA, 12/26/00	Navistar International shooting, IL, 2/5/01	Bookcliff RV Park shooting, CO, 7/3/01
Burns International Security shooting, CA, 9/8/01	Bertrand Products shooting, IN, 3/22/02	Labor Ready shooting, AL, 2/25/03
Lockheed Martin shooting, MS, 7/8/03	Windy City Core Supply shooting, IL, 8/27/03	Stateline Tavern shooting, ID, 10/24/03
ConAgra Foods Plant shooting, KS, 7/3/04	Sawyer County woods shooting, WI, 11/21/04	Damageplan show shooting, OH, 12/8/04
Fulton County Courthouse shooting, GA, 3/11/05	Living Church of God shooting, WI, 3/12/05	Red Lake Indian reservation shooting, MN, 3/21/05
Sash Assembly of God shooting, TX, 8/29/05	Postal facility shooting, CA, 1/30/06	Capitol Hill shooting, WA, 3/25/06
The Ministry of Jesus Christ shooting, LA, 5/21/06	West Nickel Mines Amish School shooting, PA, 10/2/06	Trolley Square shooting, UT, 2/12/07
Virginia Tech shooting, VA, 4/16/07	Crandon duplex shooting, WI, 10/7/07	Westroads Mall shooting, NE, 12/5/07
Youth With a Mission and New Life Church shooting, CO, 12/9/07	City council shooting, MO, 2/7/08	Northern Illinois University shooting, IL, 2/14/08
Black Road Auto shooting, CA, 3/18/08	Atlantis Plastics shooting, KY, 6/25/08	Skagit County shooting, WA, 9/2/08
Pinelake Health and Rehab Center shooting, NC, 3/29/09	Immigration services center shooting, NY, 4/3/09	Worth Street shooting, NC, 11/1/09
Army processing center shooting, TX, 11/5/09	Pierce County coffee shop shooting, WA, 11/29/09	Hot Spot Cafe shooting, CA, 4/3/10
Yoyito Cafe-Restaurant shooting, FL, 6/6/10	Hartford Beer Distributors shooting, CT, 8/3/10	City Grill shooting, NY, 8/14/10
Safeway parking lot shooting, AZ, 1/8/11	Family Law Practice shooting, AZ, 6/2/11	Forum Roller World shooting, TX, 7/23/11
IHOP shooting, NV, 9/6/11	Salon Meritage shooting, CA, 10/12/11	Su Jung Health Sauna shooting, GA, 2/21/12
Oikos University shooting, CA, 4/2/12	Café Racer shooting, WA, 5/30/12	Century 16 movie theater shooting, CO, 7/20/12
Sikh temple of Wisconsin shooting, WI, 8/5/12	Accent Signage Systems shooting, MN, 9/27/12	Sandy Hook Elementary School shooting, CT, 12/14/12
Mohawk Valley shootings, NY, 3/13/13	Pinewood Village Apartments shooting, WA, 4/21/13	Santa Monica College shooting, CA, 6/7/13
Todel Apartments shooting, FL, 7/26/13	The Washington Navy Yard shooting, DC, 9/16/2013	Cedarville Rancheria Tribal Office shooting, CA, 2/20/14
Santa Barbara County shooting, CA, 5/23/14	Marysville-Pilchuck High School shooting, WA, 10/24/14	Emanuel African Methodist Episcopal Church shooting, SC, 6/17/15
Recruiting and Naval Reserve centers shooting, TN, 7/16/15	Umpqua Community College shooting, OR, 10/1/15	Tennessee Colony campsite shooting, TX, 11/15/15
Inland Regional Center shooting, CA, 12/2/15	Cracker Barrel shooting, MI, 2/20/16	Franklin Avenue cookout shooting, PA, 3/9/16
Pulse nightclub shooting, FL, 6/12/16	Walgreens Parking Lot shooting, NV, 6/29/16	Police protest march shooting, TX, 7/7/16
Cascades Mall Macy's shooting, WA, 9/23/16	Fort Lauderdale-Hollywood International Airport shooting, FL, 1/6/17	Club 66 shooting, MS, 2/6/2017
Marathon Savings Bank shooting, WI, 3/22/17	Fiamma office shooting, FL, 6/5/17	Taos and Rio Arriba counties shooting, NM, 6/15/17
Route 91 Harvest festival shooting, NV, 10/1/17	First Baptist Church shooting, TX, 11/5/17	Rancho Tehama Elementary School shooting, CA, 11/14/17

Each entry corresponds to one of the 87 mass shootings that took place from 1999 to 2017. For each event, the table reports its location and date.The list excludes the US Territories.

Criminal Background Check System was implemented in November 1998 and allows an authorized seller to instantaneously assess whether a prospective buyer is eligible for the firearm purchase. We limited the count of background checks to purchase attempts of handgun, long gun, other (referring to frames, receivers, and other firearms that are not handguns or long guns), and multiple guns. Data were collected from 1999 to 2017 for each of the 50 States, but recordings from Connecticut and Hawaii were excluded from the analysis, since

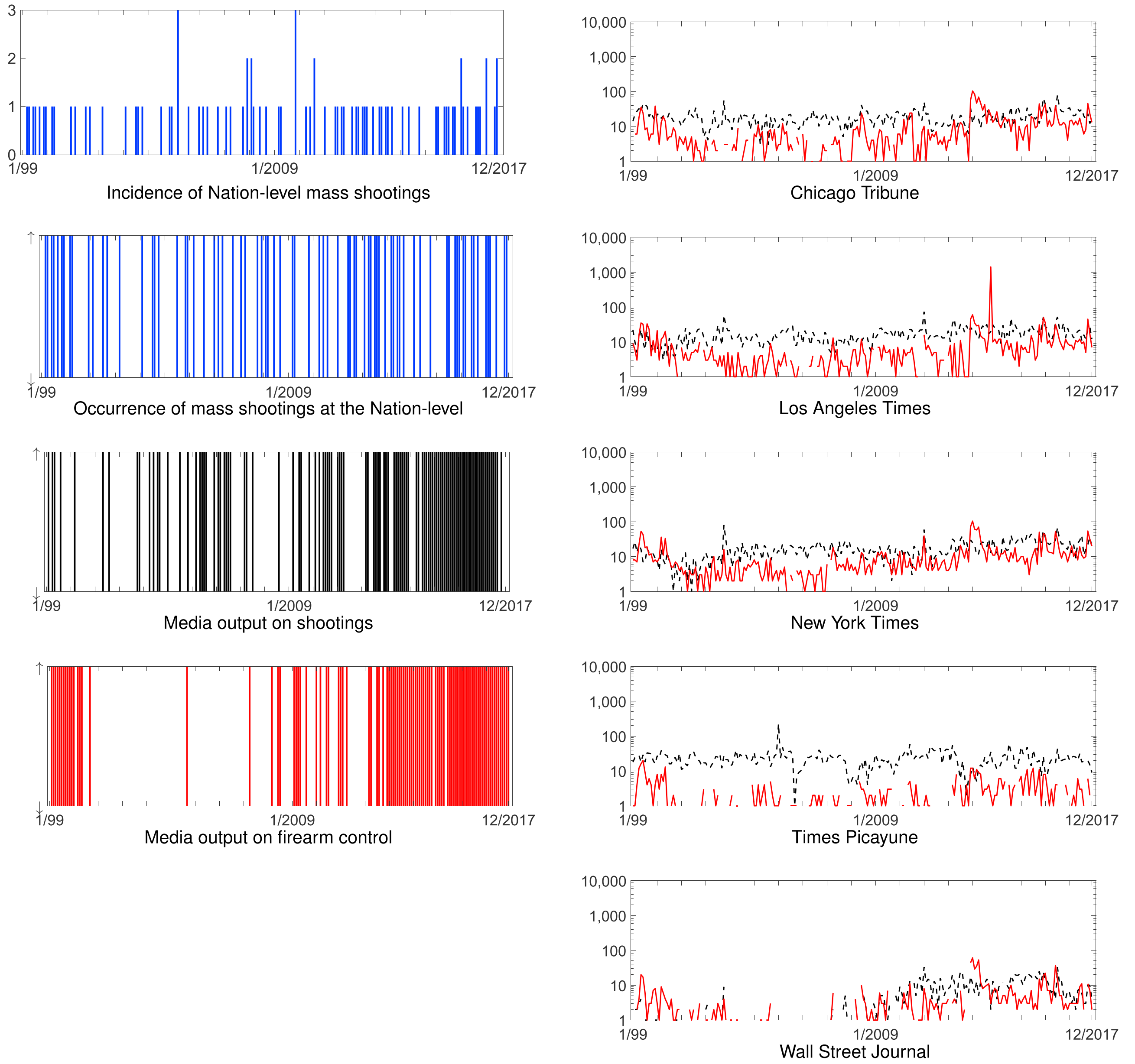

Figure 4. Synoptic Presentation of the Time-series of Mass Shootings and Media Output Used in the Three Studies
Top left panel: incidence of Nation-level mass shootings from 1999 to 2017. Right column: media output on shootings (dashed black) and firearm control (solid red) from five different newspapers from 1999 to 2017 in logarithmic scale (zero values cannot be reported). Bottom left panels: binary time-series of occurrence of mass shootings at the Nation-level, media output on shootings, and media output firearm control from 1999 to 2017; each vertical line corresponds to a ↑.

Connecticut had almost 2 years of zero background checks (possibly, because of legislation that requires all firearm transfers by licensed dealers to be processed through the Connecticut Department of Emergency Services and Public Protection[46]), and Hawaii always reported zero background checks except for 1 month in nearly 20 years (likely, because of a system of special permits in place in the State[47]).

Using the ProQuest search engine, we collected data on media coverage of shootings and firearm control. From a basic search, we identified that the two most common subjects related to "firearms" are "shootings" and "firearm laws and regulation." Hence, when searching for media coverage of shootings, we input shootings in the search toolbox, included shootings in the subject filter, and excluded firearm laws and regulation. We set the source type to "Newspapers" and specified one of five publication titles: the Chicago Tribune, Los Angeles Times, New York Times, Times Picayune, and Wall Street Journal. These daily news outlets extensively cover current events both online and offline, cater to geographically dispersed populations, and represent a wide range of opinions within the American political spectrum.[48,49] Considering one month at a time, the publication date was specified for a month between January 1999 and December 2017, and the number of results returned was manually recorded. Overall, 228 values were recorded for each of the 5 journals and a total of 18,714 documents were obtained (4,309 for the Chicago Tribune, 3,892 for the Los Angeles Times, 2,984 for the New York Times, 5,352 for the Times Picayune, and 1,177 for the Wall Street Journal). Searching for media coverage of firearm control, we performed a similar search, querying

for media coverage of firearm laws and regulation. Within the subject criterion, the firearm laws and regulations were included and none were excluded. The same source type, publication dates, and publication titles were specified. This search returned 9,106 results in total (2,169 for the Chicago Tribune, 3,040 for the Los Angeles Times, 2,442 for the New York Times, 549 for the Times Picayune, and 906 for the Wall Street Journal.) The ten time-series corresponding to media output are shown in Figure 4; statistical analysis regarding stationarity and correlation among the time-series is presented in the Supplemental Information (Tables S1 and S2).

Data Analysis

From data available on mass shootings, we compiled a binary (↓ and ↑) Nation-level time-series at a monthly resolution. A ↑ in the time-series indicated the occurrence of at least one mass shooting in a given month in any of the 50 States, while a ↓ referred to the absence of a mass shooting. In two States, no mass shooting took place (Maryland and New Jersey); in 17 States, one mass shooting took place; and the remaining States recorded up to 11 mass shootings (California). In addition to the Nation-level time-series of mass shootings (Figure 4), we also created State-level time-series that bookkept where the event occurred. The time-series of background checks for each of the States (excluding Connecticut and Hawaii) were processed as follows. First, we applied the TRAMO/SEATS method[50] to obtain seasonally adjusted and linearly detrended time-series that could be treated as stationary. Second, we transformed these continuous time-series into binary representations in which we mapped a positive value onto a ↑ and a negative (or zero) value to a ↓. For each of the five newspapers and two types of media coverage (shootings or firearm control), we performed an equivalent transformation: monthly values larger than the median were mapped onto a ↑ and values less than or equal to the median onto a ↓. For each type of media coverage, we aggregated the binary time-series from the five newspapers into a single time-series, by taking the mode (Figure 4). This preprocessing is different from Porfiri et al.,[17] whereby we did not symbolize the continuous time-series on the basis of increases or decreases between two consecutive months, but only with respect to the values relative to the median. Such an approach eases the interpretation of the transfer entropy analysis by treating all the salient time-series with the same temporal resolution of one month.

Similar to Reeping et al.,[42] States were classified as restrictive or permissive with respect to their firearm-related legal environment. Ths classification was performed by applying the k-means algorithm[23] with Euclidean metric on the values of law restrictiveness (the algorithm was applied to 48 States, excluding Connecticut and Hawaii). In all the transfer entropy computations, we systematically treated the number of background checks in permissive or restrictive States as the effect (Y process in Equation 3). For each group, we computed one transfer entropy value by taking a weighted average of the transfer entropy values according to the population of each State. We specifically computed the square of the weighted sum of the square root of transfer entropy values, divided by the sum of the square of the populations in the group—this scaling was motivated by the fact that, as a first approximation, the variance controls the value of the entropy of a random variable, as further elaborated upon in the Supplemental Information (Figure S4).

We performed three consecutive studies (Figure 1).

- In study 1, we examined the effect of the occurrence of mass shootings at the Nation-level (MS), media output on firearm control (MO_{fc}), and media output on shootings (MO_s) on the number of background checks in restrictive (BC_r) or permissive (BC_p) States to identify the main drivers underlying firearm acquisition as a function of the legal environment. Hence, for each of the two groups of States, we calculated three values of conditional transfer entropy.
- In study 2, we focused on the potential influence of the location of the mass shooting on background checks. Specifically, for each State in any of the two groups, we isolated mass shootings that occurred in that particular State (MS_{st}) from those that took place elsewhere, in any other State (MS_e). For each of the two groups of States, we ultimately computed two values of conditional transfer entropy, one measuring the potential influence of in-State mass shootings and the other being associated with the potential influence of non in-State mass shootings (occurring anywhere else in the Nation, including the States of Connecticut and Hawaii, and Washington DC).
- In study 3, we examined the influence of geographically neighboring States on the number of background checks. In this analysis, we controlled for the two variables that emerged as the main drivers of firearm acquisition from the first study (that is, the most statistically salient variables among Nation-level mass shootings, media output on firearm control, and media output on shootings). For each State in one of the two groups, we calculated the mode of the binary time-series of background checks of the neighboring States ($\overline{BC}$), similar to the approach proposed by Herrera et al.[51] to study nonlinear interactions in spatial data. By treating this time-series as a potential cause in the transfer entropy analysis, we sought to tease out the interaction between neighboring States in the Nation with respect to firearm acquisition. Such an analysis was performed by varying the number of neighbors from one to nine in steps of two, resulting in 20 values of conditional transfer entropy.

The entire statistical analysis relied on a non-parametric permutation test.[52] Specifically, to test whether a potential cause-and-effect relationships was statistically significant, we calculated a surrogate distribution of transfer entropy values by permuting the binary time-series. To preserve internal structure between the effect and the conditioning processes (Y and $Z_1, \ldots, Z_q$ in Equation 3), we proceeded as follows: (1) we held fixed the time-series of the effect and conditioning processes and (2) we permuted the time-series of the cause (X in Equation 3), by shuffling its values only among time instants corresponding to the same tuple for the effect and conditioning processes. From the surrogate distribution, we calculate a p value for the value of the corresponding conditional transfer entropy and rejected the null hypothesis of null influence with a significance level $\alpha = 0.050$. In the Supplemental Information, we illustrate the application of the approach on a synthetic dataset, demonstrating its reliability in inferring true causal links and dismissing spurious ones (Figure S5).

SUPPLEMENTAL INFORMATION

Supplemental Information can be found online at https://doi.org/10.1016/j.patter.2020.100082.

ACKNOWLEDGMENTS

This study is part of the collaborative activities carried out under the programs of the region of Murcia (Spain): “Groups of Excellence of the region of Murcia, the Fundación Séneca, Science and Technology Agency” project 19884/GERM/15 and “Call for Fellowships for Guest Researcher Stays at Universities and OPIS” project 21144/IV/19. The work of R.B.V. was funded in part by a scholarship from the Mitsui-USA foundation. M.P. would like to express his gratitude to the Technical University of Cartagena for hosting him during a Sabbatical leave and to acknowledge support from the New York University Research Challenge Fund Program. The funders had no role in study design, data collection and analysis, decision to publish, or preparation of the manuscript. The authors express their gratitude to Alain Boldini for useful discussion on the topic, to Gianluca Astudillo and Maria Grillo for assistance with the verification of the dataset, and to Jorge Ruiz Lopez for his help with the graphical abstract.

AUTHOR CONTRIBUTIONS

Conceptualization, M.P. and M.R.M.; Methodology, M.P. and M.R.M.; Software, M.R.M.; Validation, M.P. and R.B.-V.; Formal Analysis, M.P. and M.R.M.; Investigation, M.P., R.B.-V., and M.P.; Data Curation, R.B.-V.; Writing – Original Draft, M.P.; Writing – Review & Editing, M.P., R.B.-V., and M.R.M.; Visualization, M.P. and M.R.M.; Supervision, M.P.; Project Administration, M.P.; Funding Acquisition, M.P. and M.R.M.

DECLARATION OF INTERESTS

The authors declare no competing interests.

Received: April 22, 2020
Revised: May 26, 2020
Accepted: July 3, 2020
Published: August 11, 2020

REFERENCES

1. Grinshteyn, E., and Hemenway, D. (2016). Violent death rates: the US compared with other high-income OECD countries, 2010. Am. J. Med. *129*, 266–273.
2. Krouse, W.J., and Richardson, D.J. (2015). Mass Murder with Firearms: Incidents and Victims, 1999-2013 Specialist in Domestic Security and Crime Policy (Congressional Research Service). https://fas.org/sgp/crs/misc/R44126.pdf.
3. Follman, M., Aronsen, G., and Pan, D.. A guide to mass shootings in America, Mother Jones https://www.motherjones.com/politics/2012/07/mass-shootings-map/.
4. Gun Violence Archive. Past summary ledgers. https://www.gunviolencearchive.org/past-tolls.
5. Centers for Disease Control and Prevention. WISQARS™ (Web-based Injury Statistics Query and Reporting System). https://www.cdc.gov/injury/wisqars/index.html.
6. Wallace, L.N. (2015). Responding to violence with guns: mass shootings and gun acquisition. Soc. Sci. J. *52*, 156–167.
7. Studdert, D.M., Zhang, Y., Rodden, J.A., Hyndman, R.J., and Wintemute, G.J. (2017). Handgun acquisitions in California after two mass shootings. Ann. Intern. Med. *166*, 698.
8. Callcut, R.A., Robles, A.M., Kornblith, L.Z., Plevin, R.E., and Mell, M.W. (2019). Effect of mass shootings on gun sales. A 20-year perspective. J. Trauma Acute Care Surg. *87*, 531–540.
9. Liu, G., and Wiebe, D.J. (2019). A time-series analysis of firearm purchasing after mass shooting events in the United States. JAMA Netw. Open *2*, e191736.
10. Large, J. (2014). When fear outweighs reality. The Seattle Times https://www.seattletimes.com/seattle-news/when-fear-outweighs-reality.
11. Chapman University (2017). Americas top fears 2017. The Seattle Times https://blogs.chapman.edu/wilkinson/2017/10/11/americas-top-fears-2017.
12. Aisch, G., and Keller, J. (2016). What happens each time after call for new gun restrictions? Sales go up. The New York Times http://www.nytimes.com/interactive/2015/12/10/us/gun-sales-terrorism-obama-restrictions.html.
13. Kegley, J. (2015). Gun rush: weapons sales soar in Kentucky on threat of new federal restrictions. Lexington Herald Leader https://www.kentucky.com/news/local/crime/article44398467.html.
14. Naik, R. (2016). Heres Why Gun Stocks Rise after Mass Shootings (CNN Money). https://money.cnn.com/video/news/2017/10/03/gun-stock-sales-rise.cnnmoney/index.html.
15. Smith, A. (2017). How President Trump Is Bad for the Gun Industry (CNN Money). http://money.cnn.com/2017/02/03/news/companies/trump-gun-ammo-sales/.
16. Stroebe, W., Leander, N.P., and Kruglanski, A.W. (2017). The impact of the Orlando mass shooting on fear of victimization and gun-purchasing intentions: not what one might expect. PLoS One *12*, e0182408.
17. Porfiri, M., Sattanapalle, R.R., Nakayama, S., Macinko, J., and Sipahi, R. (2019). Media coverage and firearm acquisition in the aftermath of a mass shooting. Nat. Hum. Behav. *3*, 913–921.
18. Bossomaier, T., Barnett, L., Harr, M., and Lizier, J.T. (2016). An Introduction to Transfer Entropy: Information Flow in Complex Systems (Springer).
19. Granger, C.W.J. (1969). Investigating causal relations by econometric models and cross-spectral methods. Econometrica *37*, 424–438.
20. Wiener, N. (1956). The theory of prediction. In Modern Mathematics for Engineers, E.F. Beckenbach and R. Weller, eds. (McGraw-Hill Book Company, Inc.), pp. 125–139.
21. Abaid, N., Macinko, J., Silver, D., and Porfiri, M. (2015). The effect of geography and citizen behavior on motor vehicle deaths in the United States. PLoS One *10*, e0123339.
22. Gallos, L.K., Barttfeld, P., Havlin, S., Sigman, M., and Makse, H.A. (2012). Collective behavior in the spatial spreading of obesity. Sci. Rep. *2*, 1–9.
23. Wu, J. (2012). Advances in K-Means Clustering: A Data Mining Thinking (Springer).
24. Institute of Medicine, National Research Council (2013). Priorities for Research to Reduce the Threat of Firearm-Related Violence (The National Academies Press).
25. Banerjee, A., Chitnis, U., Jadhav, S., Bhawalkar, J., and Chaudhury, S. (2009). Hypothesis testing, type I and type II errors. Ind. Psychiatry J. *18*, 127–131.
26. Walker, J.L. (1969). The diffusion of innovations among the American states. Am. Polit. Sci. Rev. *63*, 880–899.
27. Butz, A.M., Fix, M.P., and Mitchell, J.L. (2015). Policy learning and the diffusion of stand-your-ground laws. Polit. Policy *43*, 347–377.
28. Fay, D.L., and Wenger, J.B. (2016). The political structure of policy diffusion. Policy Stud. J. *44*, 349–365.
29. Macinko, J., and Silver, D. (2012). Improving state health policy assessment: an agenda for measurement and analysis. Am. J. Public Health *102*, 1697–1705.
30. Luca, M., Malhotra, D., and Poliquin, C. (2020). The impact of mass shootings on gun policy. J. Public Econ. *181*, 104083.
31. Towers, S., Gomez-Lievano, A., Khan, M., Mubayi, A., and Castillo-Chavez, C. (2015). Contagion in mass killings and school shootings. PLoS One *10*, e0117259.
32. Duwe, G. (2020). Patterns and prevalence of lethal mass violence. Criminol. Public Policy *19*, 17–35.
33. Huff-Corzine, L., and Corzine, J. (2020). The devil's in the details: measuring mass violence. Criminol. Public Policy *19*, 317–333.
34. Croitoru, A., Kien, S., Mahabir, R., Radzikowski, J., Crooks, A., Schuchard, R., Begay, T., Lee, A., Bettios, A., and Stefanidis, A. (2020). Responses to mass shooting events: the interplay between the media and the public. Criminol. Public Policy *19*, 335–360.
35. Bressler, S.L., and Seth, A.K. (2011). Wiener–Granger causality: a well established methodology. Neuroimage *58*, 323–329.
36. Vicente, R., Wibral, M., Lindner, M., and Pipa, G. (2011). Transfer entropy a model-free measure of effective connectivity for the neurosciences. J. Comput. Neurosci. *30*, 45–67.
37. Runge, J., Bathiany, S., Bollt, E., Camps-Valls, G., Coumou, D., Deyle, E., Glymour, C., Kretschmer, M., Mahecha, M.D., Muñoz-Marí, J., et al. (2019). Inferring causation from time series in earth system sciences. Nat. Commun. *10*, 1–13.
38. Porfiri, M. (2018). Inferring causal relationships in zebrafish-robot interactions through transfer entropy: a small lure to catch a big fish. Anim. Behav. Cogn. *5*, 341–367.
39. Barnett, L., Barrett, A.B., and Seth, A.K. (2009). Granger causality and transfer entropy are equivalent for Gaussian variables. Phys. Rev. Lett. *103*, 238701.
40. Marinazzo, D., Pellicoro, M., and Stramaglia, S. (2012). Causal information approach to partial conditioning in multivariate data sets. Comput. Math. Methods Med. *2012*, 303601.
41. State Firearm Laws project. National data. https://statefirearmlaws.org/national-data.
42. Reeping, P.M., Cerdá, M., Kalesan, B., Wiebe, D.J., Galea, S., and Branas, C.C. (2019). State gun laws, gun ownership, and mass shootings in the US: cross sectional time series. BMJ *364*, l542.

CellPress
OPEN ACCESS

43. Berkowitz, B., Lu, D., and Alcantara, C.. Mass shooting statistics in the United States. https://www.washingtonpost.com/graphics/2018/national/mass-shootings-in-america/.
44. Inter-University Consortium for Political and Social Research (2015). Uniform Crime Reporting Program Data: Supplementary Homicide Reports (United States Department of Justice, Federal Bureau of Investigation).
45. National Instant Criminal Background Check System. National data. https://fbi.gov/services/cjis/nics/nics.
46. Giffords Law Center to Prevent Gun Violence. Background check procedures in Connecticut. https://lawcenter.giffords.org/background-check-procedures-in-connecticut/.
47. Giffords Law Center to Prevent Gun Violence. Background check procedures in Hawaii. https://lawcenter.giffords.org/background-check-procedures-in-hawaii/.
48. Gentzkow, M., and Shapiro, J.M. (2010). What drives media slant? Evidence from US daily newspapers. Econometrica *78*, 35–71.
49. Ho, D.E., and Quinn, K.M. (2008). Measuring explicit political positions of media. Quart. J. Polit. Sci. *3*, 353–377.
50. Hood, C.C., Ashley, J.D., and Findley, D.F. (2000). An empirical evaluation of the performance of TRAMO/SEATS on simulated series. In Proceedings of the Business and Economics Section https://www.census.gov/content/dam/Census/library/working-papers/2000/adrm/asa00-ts.pdf.
51. Herrera, M., Mur, J., and Ruiz, M. (2016). Detecting causal relationships between spatial processes. Pap. Reg. Sci. *95*, 577–594.
52. Runge, J. (2018). Causal network reconstruction from time series: from theoretical assumptions to practical estimation. Chaos *28*, 075310.

PATTER, Volume *1*

Supplemental Information

Self-Protection versus Fear of Stricter Firearm Regulations: Examining the Drivers of Firearm Acquisitions in the Aftermath of a Mass Shooting

Maurizio Porfiri, Roni Barak-Ventura, and Manuel Ruiz Marín

Summary: Supplementary Information contains legal environment and population data, a year-by-year cluster analysis of States, a comparison between different definitions of firearm-related legal environments, statistical details about the time-series of media output, and the validation of the approach on a synthetic data set.

S1. Legal environment and population data

Figure S1 shows data on law restrictiveness and population in the form of two histograms. Note that the two histograms are independently ordered according to the value of the variable they represent.

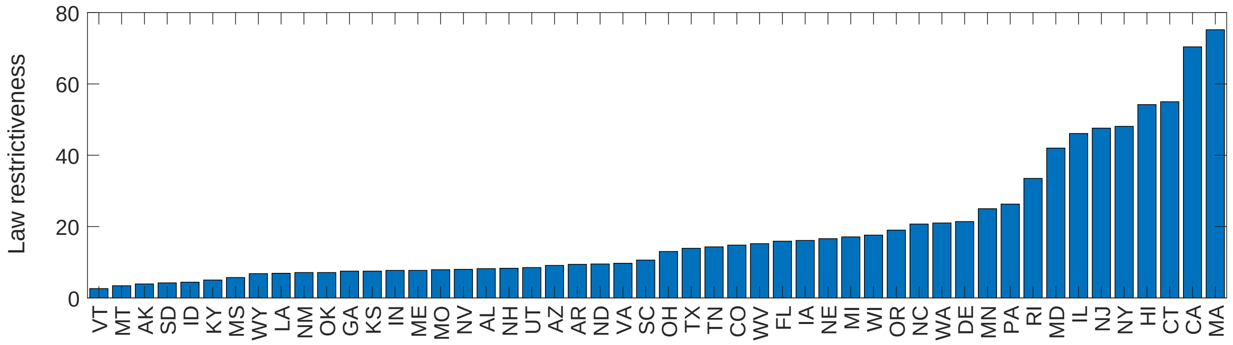

(a) Legal environment

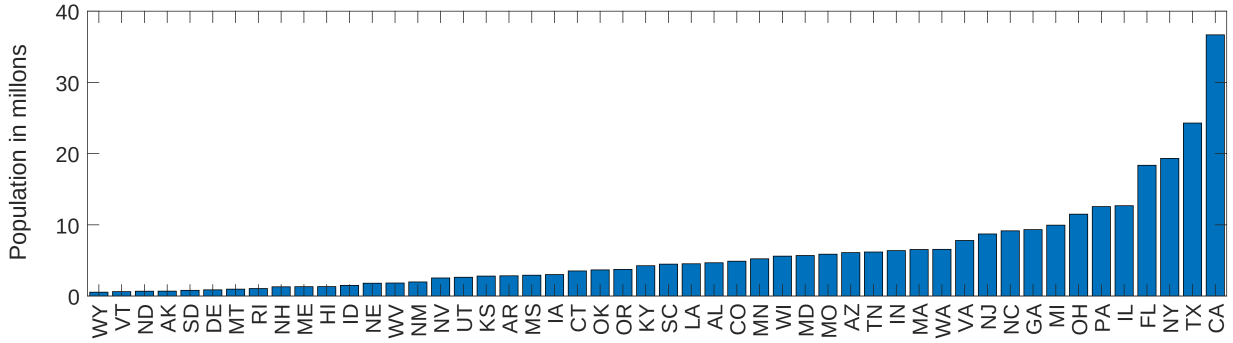

(b) Population

Figure S1: Law restrictiveness index, scored from 0% to 100%, and population in the US, averaged from 1999 to 2017.

S2. Year-by-year cluster analysis of States

Figure S2 shows the time-series of law restrictiveness for the 48 States included in our study, scored as the fraction of the 133 firearm safety laws that were in effect at any given year between 1999 and 2017. The time-series of each State is colored according to whether the State was scored as restrictive (red) or permissive (green) from the analysis in the main text, which was based on the average fraction of these laws that were in effect from 1999 to 2017.

As evident from the figure, changes in the legal environment across the Nation are secondary, whereby the time-series are always clustered in two groups. More precisely, the application of the k-means clustering algorithm year-by-year indicated that States group equivalently to the main text from 1999 to 2010. From 2011 to 2017, the algorithm would assign Rhode Island to the permissive group. Notably, the population of

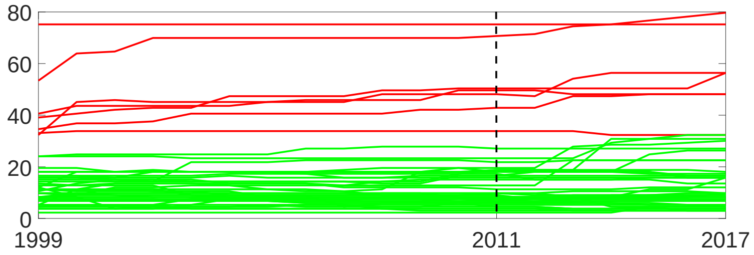

Figure S2: Time-series of law restrictiveness from 1999 to 2017, for the 48 States considered in the main text (all States, excluding Connecticut and Hawaii). Time-series are color coded according to their original assignment to the restrictive (red) or permissive (green) groups. The year 2011 is marked with a dashed line to indicate that a cluster analysis performed in any year in the range 2011 $-$ 2017 would lead to Rhode Island be assigned to the permissive group.

Rhode Island is much smaller than any the other State in the restrictive group, thereby causing a negligible change in the fraction of the population living in restrictive versus permissive States.

S3. Comparison between definitions of legal environments

We compared our definition of law restrictiveness with the one proposed by Reeping et al.,[1] which was based on the 1998-2015 edition of the Traveler's Guide to the Firearms Laws of the Fifty States. This report serves as a reference guide for firearm owners traveling between States: States with a rating of zero are completely restrictive and those with rating of one hundred are completely permissive. As detailed by Reeping et al.,[1] the conditions that the report takes into consideration to rate the States are more than 13, spanning from standard firearms ownership and permit requirements to whether it is possible to keep a firearm in one's vehicle at colleges and K-12 schools.

Figure S3 compares our metric of law restrictiveness of the firearm-related legal environment of a State (horizontal axis) with the score by Reeping et al.[1] (vertical axis), extracted from their supplementary figure by manually taking the medians of each of the boxplots. Excellent agreement between the metrics can be evidenced from the data, with an R^2 of 0.821.

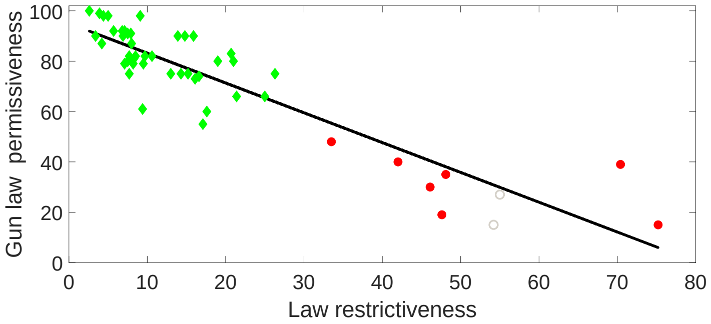

Figure S3: Comparison between the proposed measure of law restrictiveness (from 0% to 100%; 0% being completely permissive and 100% completely restrictive) and the law permissiveness score proposed by Reeping et al.[1] (from 0 to 100; 0 being completely restrictive and 100 completely permissive). Each marker corresponds to one of the 50 States and the color-coding is the same as in the main text (red circle: one of the seven States in the restrictive group; green diamond: one of the 41 States in the permissive group; and open grey circle: one of the two States that are excluded from the study due to lack of data). A regression line is also shown for clarity.

S4. Further statistical analysis of media output

Here, we provide further statistical details regarding the ten time-series of media coverage from the selected newspapers (Chicago Tribune, Los Angeles Times, New York Times, Times Picayune, and Wall Street Journal). First, we confirmed that the time-series could be treated as stationary by performing the augmented Dickey–Fuller test on each of them. The results of the analysis rejected the null hypothesis of unit root with $p < 0.001$.

Second, we examined the correlation between each pair of time-series pertaining to the same type of media coverage. Tables S1 and S2 report the values of the Pearson correlation coefficients for each of pair of media coverage of shootings and firearm control, respectively. Although all the coefficients are positive, they vary over a wide range from 0.007 to 0.899, suggesting that the selected newspapers contribute different levels of coverage of shootings and mass shootings. This is especially evident when examining media output on firearm control, in which both the Los Angeles Times and Times Picayune from California and Louisiana, respectively, neither correlate with each other nor with any other newspaper.

	Chicago Tribune	Los Angeles Times	New York Times	Times Picayune	Wall Street Journal
Chicago Tribune	1.000	0.644	0.873	0.106	0.899
Los Angeles Times	-	1.000	0.580	0.054	0.572
New York Times	-	-	1.000	0.134	0.876
Times Picayune	-	-	-	1.000	0.069
Wall Street Journal	-	-	-	-	1.000

Table S1: Pearson correlation coefficient between the time-series on media coverage of firearm control for the five newspapers.

	Chicago Tribune	Los Angeles Times	New York Times	Times Picayune	Wall Street Journal
Chicago Tribune	1.000	0.035	0.503	0.644	0.641
Los Angeles Times	-	1.000	0.007	0.030	0.031
New York Times	-	-	1.000	0.476	0.398
Times Picayune	-	-	-	1.000	0.624
Wall Street Journal	-	-	-	-	1.000

Table S2: Pearson correlation coefficient between the time-series on media coverage of shootings for the five newspapers.

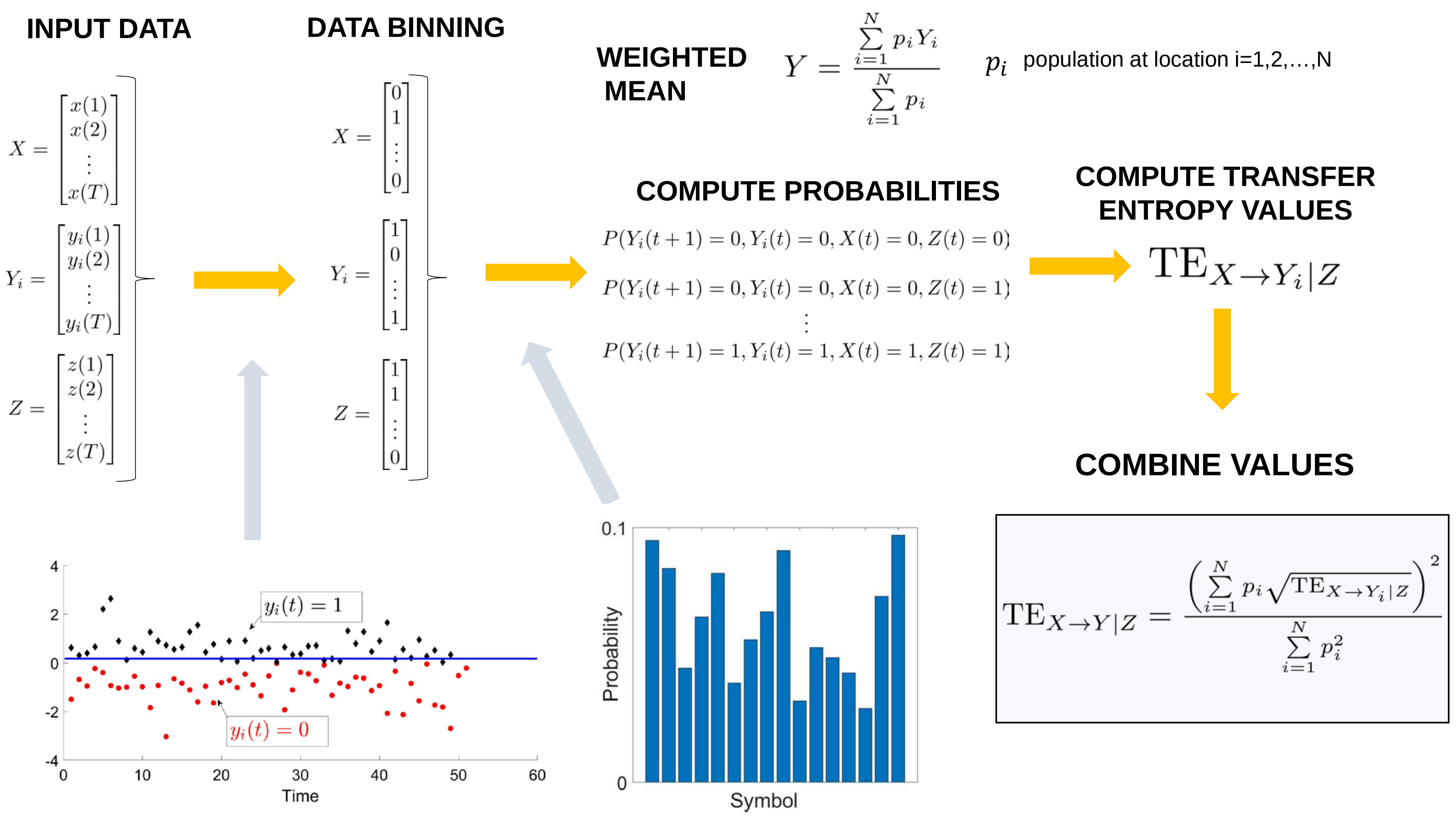

Figure S4: Sketch of the steps needed for transfer entropy computation. Starting from the time-series of all the processes of length $T = 228$, we compute binary versions by binning the data. Next, we estimate joint probability mass functions probabilities by simply counting the ones and the zeros, from which we calculate transfer entropy values for $i = 1, \ldots, N$, with $N = 10$. Finally, the population values are utilized to calculate transfer entropy.

S5. Demonstration of the approach on synthetic data

To demonstrate the performance of the proposed causality analysis based on transfer entropy, we simulated a controlled experiment with synthetic data. Specifically, we considered the following linear autoregressive model, where X is the cause (similar to any of the media output or occurrence of mass shootings at the National level), Y is the effect, sampled at $N = 10$ different locations (similar to a pro capita background check at the State-level), and Z is a third independent process (acting as a potential confound):

$$X(t+1) = \alpha_X X(t) + \gamma_{XZ} Z(t) + \varepsilon_X \tag{S1a}$$

$$Y_i(t+1) = \alpha_Y Y_i(t) + \beta\gamma_{YXi} X(t) + \varepsilon_{Yi}, \quad i = 1, \dots N \tag{S1b}$$

where $\alpha_Y = 0.2$, $\alpha_X = 0.1$, $\gamma_{XZ} = 0.2$, $\gamma_{YXi} = 0.1$ for $i = 1, \dots, N/2$ and $\gamma_{YXi} = 0.3$ for $i = N/2+1, \dots, N$, and Z, ε_X, ε_{Yi}, with $i = 1, \dots, N$, are normal independent identically distributed with zero mean and unit variance, that is, $\mathcal{N}(0, 1)$. The parameter β is used to tune the strength of the interaction.

S5.1. Inference of causal relationships

For each value of β from 0.1 to 2 with steps of 0.1, we simulated 100 processes through Eq. (S1) for 228 time-steps, equal to the length of the available time-series. For each simulation, we estimated the weighted sum of the square root of transfer entropy values $\mathrm{TE}_{X\to Y_i|Z}$ and $\mathrm{TE}_{Z\to Y_i|X}$, for $i = 1, \dots, N$, divided by the sum of the square of the populations. In the estimation, we assumed for each of the ten locations the following populations: $\{p_1 = 0.1, p_2 = 0.2, p_3 = 0.3, p_4 = 0.4, p_5 = 0.5, p_6 = 0.6, p_7 = 0.7, p_8 = 0.8, p_9 = 0.9, p_{10} = 1.0\}$. Fig. S4 illustrates the steps of the transfer entropy computation.

Figure S5 summarizes the accuracy of the inferences for 100 simulations. Therein, we specifically report the following quantities: true and false positive rates of rejections of the null hypothesis of no causality from X to Y, and true and false positive rates of failing to reject the null hypothesis of no causality from Z to Y. The permutation tests were carried out using 1,000 bootstrap realizations at a confidence level $\alpha = 0.050$. For example, a true positive rate of 90 on the causal link from X to Y indicates that in 90 out of 100 simulations, we rejected the hypothesis of no causality (that is, $p < 0.050$ in permutation tests). Similarly, a true positive rate of 90 on the causal link from Z to Y indicates that in 90 out of 100 simulations, we failed to reject the hypothesis of no causality (that is, $p \geq 0.050$ in permutation tests).

The results of the inference in Figure S5 offer compelling evidence in favor of the feasibility of the proposed approach to unfold causal relationships: not only is the method able to exactly discover the true causal link from X to Y for sufficiently large values of the coupling β (black markers), but also it is able to filter out the spurious interaction between X and Y for any choice of the parameter β (red markers).

S5.2. Verification of the weighting scheme for transfer entropy values

Next, we utilize the same example to illustrate the mathematical rationale for the chosen weighting scheme for transfer entropy values corresponding to the effect variables Y_i, for $i, \dots, N$. In the model in Eq. (S1), the effect variables represent pro capita background checks. Hence, computing the overall effect in the entire population requires multiplying each of them by the corresponding population. The resulting, normalized, variable is $Y = \left(\sum_{i=1}^{N} p_i Y_i\right) / \left(\sum_{i=1}^{N} p_i\right)$. Based on this aggregated variable, we calculate $\mathrm{TE}_{X\to Y|Z}$, which can be exactly computed from the classical expression for the entropy of a normal variable.[2] By recalling that the (differential) entropy of a normal variable G with variance σ_G^2 is $H(G) = \frac{1}{2}\log_2(2\pi e\sigma_G^2)$, where e is Nepero's number, we establish

$$\mathrm{TE}_{X\to Y|Z} = \frac{1}{2}\log_2\left(1 + \frac{\left(\sum_{i=1}^{N} p_i\gamma_{YXi}\right)^2 \sigma_X^2}{\sum_{i=1}^{N} p_i^2}\right) \tag{S2a}$$

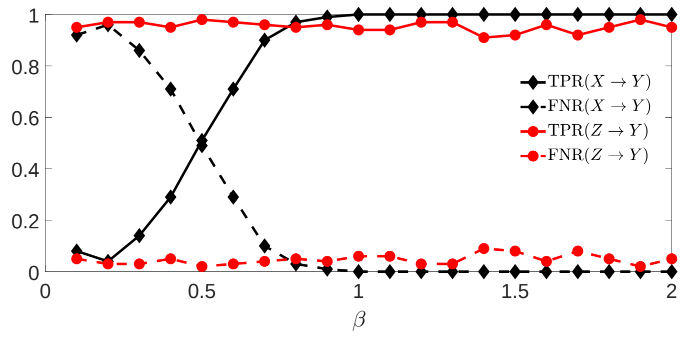

Figure S5: Results of the inference for a simulated data set, generated using Eq. (S1) and parameters reported in the text. Accuracy is assessed in terms of the rejection of the null hypotheses of no causality from X to Y and from Z to Y over 100 simulations. Specifically, we present the true positive rate of rejections of the null hypothesis of no causality from X to Y, $\mathrm{TPR}(X \to Y)$ (black solid line and diamond-shaped markers); false negative rate of rejections of the null hypothesis of no causality from X to Y, $\mathrm{FNR}(X \to Y) = 1 - \mathrm{TPR}(X \to Y)$ (black dashed line and diamond-shaped markers); true positive rate of failing to reject the null hypothesis of no causality from Z to Y, $\mathrm{TPR}(Z \to Y)$ (red solid line and circular markers); and false negative rate of failing to reject the null of hypothesis of no causality from Z to Y, $\mathrm{FNR}(Z \to Y) = 1 - \mathrm{TPR}(Z \to Y)$ (red dashed line and circular markers). The permutation tests were carried out using 1,000 bootstrap realizations at a confidence level $\alpha = 0.050$.

$$\mathrm{TE}_{X \to Y_i|Z} = \frac{1}{2} \log_2 \left(1 + \gamma_{YXi}^2 \sigma_X^2\right) \tag{S2b}$$

By multiplying both sides of the equations above by 2, taking the power of both sides, and carrying out a MacLaurin expansion for small values of transfer entropy, we obtain

$$\mathrm{TE}_{X \to Y|Z} \approx \frac{\left(\sum_{i=1}^{N} p_i \sqrt{\mathrm{TE}_{X \to Y_i|Z}}\right)^2}{\sum_{i=1}^{N} p_i^2} \tag{S3}$$

By applying the well-known Cauchy-Schwartz inequality, the numerator in Eq. (S3) can be bounded by $\left(\sum_{i=1}^{N} p_i^2\right)\left(\sum_{i=1}^{N} \mathrm{TE}_{X \to Y_i|Z}\right)$, such that

$$\mathrm{TE}_{X \to Y|Z} \lessapprox \sum_{i=1}^{N} \mathrm{TE}_{X \to Y_i|Z} \tag{S4}$$

This expression suggests that the value of transfer entropy in Eq. (S3) scales with N. Throughout the tables in the main text, we observe that transfer entropy values for restrictive States are, in fact, always a fraction of the corresponding values for permissive States.

REFERENCES

1. Reeping, P.M., Cerdá, M., Kalesan, B., Wiebe, D.J., Galea, S., Branas, C.C., (2019). State Gun Laws, Gun Ownership, and Mass Shootings in the US: Cross Sectional Time Series. BMJ 364, l542, `https://doi.org/10.1136/bmj.l542`.
2. Cover, T.M., and Thomas, J.A. (2006). Elements of Information Theory. (Wiley Series in Telecommunications and Signal Processing).
